# Safety and Efficacy of Liraglutide, 3.0 mg, Once Daily vs Placebo in Patients With Poor Weight Loss Following Metabolic Surgery: The BARI-OPTIMISE Randomized Clinical Trial

**DOI:** 10.1001/jamasurg.2023.2930

**Published:** 2023-07-26

**Authors:** Jessica Mok, Mariam O. Adeleke, Adrian Brown, Cormac G. Magee, Chloe Firman, Christwishes Makahamadze, Friedrich C. Jassil, Parastou Marvasti, Alisia Carnemolla, Kalpana Devalia, Naim Fakih, Mohamed Elkalaawy, Andrea Pucci, Andrew Jenkinson, Marco Adamo, Rumana Z. Omar, Rachel L. Batterham, Janine Makaronidis

**Affiliations:** 1 Division of Medicine, University College London Centre for Obesity Research, Rayne Institute, London, United Kingdom; 2 Bariatric Centre for Weight Management and Metabolic Surgery, University College London Hospitals, National Health Service Foundation Trust, London, United Kingdom; 3 National Institute for Health and Care Research, University College London Hospitals Biomedical Research Centre, London, United Kingdom; 4 Department of Statistical Science, University College London, London, United Kingdom; 5 Bariatric Surgery Department Homerton University Hospital National Health Service Trust, London, United Kingdom

## Abstract

**Importance:**

Metabolic surgery leads to weight loss and improved health, but these outcomes are highly variable. Poor weight loss is associated with lower circulating levels of glucagon-like peptide-1 (GLP-1).

**Objective:**

To assess the efficacy and safety of the GLP-1 receptor agonist, liraglutide, 3.0 mg, on percentage body weight reduction in patients with poor weight loss and suboptimal GLP-1 response after metabolic surgery.

**Design, Setting, and Participants:**

The Evaluation of Liraglutide 3.0 mg in Patients With Poor Weight Loss and a Suboptimal Glucagon-Like Peptide-1 Response (BARI-OPTIMISE) randomized placebo-controlled trial recruited adult patients at least 1 year after metabolic surgery who had experienced 20% or less body weight loss from the day of surgery and a suboptimal nutrient-stimulated GLP-1 response from 2 hospitals in London, United Kingdom, between October 2018 and November 2019. Key exclusion criteria were type 1 diabetes; severe concomitant psychiatric, gastrointestinal, cardiac, kidney or metabolic disease; and use of insulin, GLP-1 receptor analogues, and medication that can affect weight. The study period was 24 weeks followed by a 4-week follow-up period. Last participant follow-up was completed in June 2020. All participants and clinical study personnel were blinded to treatment allocation. Of 154 assessed for eligibility, 70 met trial criteria and were included in the study, and 57 completed follow-up.

**Interventions:**

Liraglutide, 3.0 mg, once daily or placebo as an adjunct to lifestyle intervention with a 500-kcal daily energy deficit for 24 weeks, on a 1:1 allocation by computer-generated randomization sequence, stratified by surgery type (Roux-en-Y gastric bypass [RYGB] or sleeve gastrectomy [SG]) and type 2 diabetes status.

**Main Outcome and Measures:**

The primary outcome was change in percentage body weight from baseline to the end of the 24-week study period based on an intention-to-treat analysis. Participant safety was assessed through monitoring of biochemical parameters, including kidney and liver function, physical examination, and assessment for adverse events.

**Results:**

A total of 70 participants (mean [SD] age, 47.6 [10.7] years; 52 [74%] female) with a poor weight loss response following RYGB or SG were randomized to receive 3.0-mg liraglutide (n = 35) or placebo (n = 35). All participants received at least 1 dose of the trial drug. Eight participants discontinued treatment (4 per group), and 2 in the 3.0-mg liraglutide group and 1 in the placebo group were lost to follow-up. Due to COVID-19 restrictions, 3 participants in the 3.0-mg liraglutide group and 7 in the placebo group were unable to attend their final in-person assessment. Estimated change in mean (SD) percentage body weight from baseline to week 24 was −8.82 (4.94) with liraglutide, 3.0 mg (n = 31), vs −0.54 (3.32) with placebo (n = 26). The mean difference in percentage body weight change for liraglutide, 3.0 mg, vs placebo was −8.03 (95% CI, −10.39 to −5.66; *P* < .001). Adverse events, predominantly gastrointestinal, were more frequent with liraglutide, 3.0 mg (28 events [80%]), than placebo (20 events [57%]). There were no serious adverse events and no treatment-related deaths.

**Conclusion and Relevance:**

These findings support the use of adjuvant liraglutide, 3.0 mg, for weight management in patients with poor weight loss and suboptimal GLP-1 response after metabolic surgery.

**Trial Registration:**

ClinicalTrials.gov Identifier: NCT03341429

## Key Points

**Question:**

Is liraglutide, 3.0 mg, once daily safe and effective for weight management in patients with poor weight loss following metabolic surgery?

**Findings:**

In the BARI-OPTIMISE randomized clinical trial including 70 patients with poor weight loss and suboptimal nutrient-stimulated glucagon-like peptide-1 response following metabolic surgery, those randomized to 3.0-mg liraglutide once daily had a significantly greater reduction in body weight compared to those randomized to placebo.

**Meaning:**

The results of this study demonstrate that liraglutide, 3.0 mg, was safe and effective as a weight management intervention in this patient population.

## Teaser

This randomized clinical trial evaluates the safety and efficacy of 3.0-mg liraglutide vs placebo in body weight reduction among patients with poor weight loss and suboptimal glucagon-like peptide-1 response after metabolic surgery.

## Introduction

Metabolic surgery is the most effective known treatment option for people with severe obesity, leading to marked sustained weight loss, improvement or remission of obesity-associated comorbidities, improved quality of life, and reduced all-cause mortality.^1,2,3^ While on a population level metabolic surgery is highly effective, on an individual level the response is highly variable.^4,5^ This variability impacts health as postoperative improvement or resolution of obesity-associated comorbidities are associated with weight loss.^6,7,8^ Poor weight loss or weight regain, resulting in less than 20% weight loss, affect up to 1 in 4 patients who undergo metabolic surgery.^8,9^ There is an unmet clinical need for effective therapeutic strategies for these patients.

Metabolic surgery alters gastrointestinal signals that regulate energy and glucose homeostasis.^10^ In most patients, metabolic surgery results in supraphysiological nutrient-stimulated circulating levels of the satiety gut hormone glucagon-like peptide-1 (GLP-1).^11,12^ However, studies undertaken in patients with poor vs good postsurgery weight loss demonstrate that individuals with poor weight loss have increased appetite coupled with an unfavorable postoperative gut hormone profile, including lower circulating GLP-1 levels.^10,11^ Treatment with GLP-1 analogs may therefore benefit people with poor postsurgery weight loss.

The GLP-1 Receptor Agonist Intervention for Poor Responders After Bariatric Surgery (GRAVITAS) randomized clinical trial^13^ undertaken in individuals with persistent or recurrent type 2 diabetes after metabolic surgery using the type 2 diabetes dose (1.8 mg) liraglutide, as an adjunct to a lifestyle intervention, showed improved glycemic control with a mean weight difference of −4.2 kg (95% CI, −6.8 to −1.4) after 26 weeks. Several nonrandomized studies using 3.0-mg liraglutide, the weight-management dose, in people with insufficient weight loss following metabolic surgery have also reported weight reduction.^7,14,15^ However, to our knowledge, there are no randomized clinical trials examining the efficacy and safety of liraglutide, 3.0 mg, in this patient group. Thus, the aim of the Evaluation of Liraglutide 3.0 mg in Patients With Poor Weight Loss and a Suboptimal Glucagon-Like Peptide-1 Response (BARI-OPTIMISE) trial was to confirm superiority of liraglutide, 3.0 mg, compared to placebo on percentage body weight reduction, as an adjunct to a lifestyle intervention (500-kcal deficit), in individuals with poor weight loss after sleeve gastrectomy (SG) or Roux-en-Y gastric bypass (RYGB) with a suboptimal GLP-1 response. The BARI-OPTIMISE trial also aimed to determine whether 24 weeks of liraglutide, 3.0 mg, caused greater reduction in adiposity, improvement in metabolic indices, physical function, and health-related quality of life than placebo.

## Methods

### Study Design

In BARI-OPTIMISE, a double-blinded, randomized, placebo-controlled, parallel group trial, we recruited patients with poor weight loss and a suboptimal nutrient-stimulated GLP-1 response at least 12 months following primary RYGB or SG. The trial was undertaken at University College London Hospital (UCLH). Participants were recruited from UCLH and Homerton University Hospital, London, UK. The study protocol and statistical analysis plan are included in Supplement 1. Written informed consent was obtained from all participants. The trial was approved by London-Dulwich Research Ethics Committee (187/LO/00300238) and was conducted in accordance with the Declaration of Helsinki, the principles of Good Clinical Practice and all applicable regulatory requirements, including the Research Governance Framework and the Medicines for Human Use (Clinical Trial) Regulations 2004 and any subsequent amendments. The trial was registered at ClinicalTrials.gov (NCT03341429), the UK Medicines and Healthcare products Regulatory Agency (MHRA), and the European Union Drug Regulating Authority Clinical Trials (EudraCT) (EudraCT Number 2017-002407-10). The study followed the Consolidated Standards of Reporting Trials (CONSORT) reporting guideline.

Poor weight loss was defined as 20% or less total body weight loss from the day of surgery. Circulating GLP-1 levels were measured in the fasted state and 30 minutes following a 500-kcal test meal. Suboptimal GLP-1 response was defined as a 2-fold or less increase in circulating active GLP-1 between 0 and 30 minutes following the meal. This cutoff was based on a previous study^12^ by our group where meal-stimulated GLP-1 responses were assessed following RYGB and SG.

### Participants

Participants were screened between September 14, 2018, and October 29, 2019, and commenced treatment between October 24, 2018, and November 28, 2019. Key exclusion criteria were type 1 diabetes; severe psychiatric disease; severe kidney, liver, or cardiovascular disease; inflammatory bowel disease, history of pancreatitis; gastroparesis; concomitant use of dipeptidyl peptidase IV–inhibitors, GLP-1–receptor agonists, insulin, or other medications that affect weight; pregnancy; and breastfeeding. Full eligibility criteria are in the study protocol in Supplement 1.

### Meal-Stimulated Active GLP-1

Fasting baseline blood samples and subjective appetite (assessed using validated visual analog scores) assessments were obtained, followed by a standardized 500-kcal liquid meal, consumed within 10 minutes. Blood samples and appetite assessments were retaken 30 minutes after the start of the meal.

### Randomization

Eligible participants were randomly assigned (1:1) to either liraglutide, 3.0 mg (Novo Nordisk), or placebo (saline solution), via self-administered once daily subcutaneous injections with identical-appearing pens. All participants and clinical study personnel were blinded. Randomization was carried out by a computer-generated randomization sequence (Sealed Envelope) stratified by type of surgery (RYGB or SG) and type 2 diabetes status.

### Procedures

Prior to commencing treatment, baseline assessments were performed, including sociodemographic data collection and medical history. Participant race and ethnicity were self-reported in response to a question asked by the investigator. This information was captured in order to accurately describe the demographic characteristics of the study population. Participants were instructed to dose escalate over the first 4 weeks, starting with 0.6 mg once daily and increasing by 0.6 mg weekly until 3.0 mg was reached at week 5 (eFigure 1 in Supplement 2). Participants in the intervention and placebo groups followed an identical dose escalation protocol. Between week 4 and week 24, participants administered 3.0 mg or their maximum tolerated dose daily. All participants received dietary and lifestyle counseling aiming for a daily 500-kcal energy deficit at baseline and weeks 2, 4, 8, and 17 and were encouraged to undertake a minimum of 150 minutes weekly moderate to vigorous exercise.

### Outcomes

The primary end point was percentage change in body weight from baseline to week 24. Body weight was measured using a weighing scale (Tanita DC-430MAS) with participants wearing light clothes and no shoes to the nearest 0.1 kg. Percentage body weight loss was calculated as 100 × [(body weight at baseline − body weight at week 24) / body weight at baseline].

Secondary outcomes included proportion of patients who lost at least 5% of their baseline body weight, change in body weight (kg) from baseline and change in body composition (fat mass, lean muscle mass, and bone mineral density) assessed using a whole-body dual energy X-ray absorptiometry (Discovery A DXA system version 13.4.2). Metabolic secondary outcomes included change from baseline in glycemic indices (fasting glucose, fasting insulin, and hemoglobin A_1c_), lipids (fasting total cholesterol, low-density lipoprotein cholesterol, high-density lipoprotein cholesterol, and triglycerides), and C-reactive protein. Physical activity and physical function were assessed at baseline and end of treatment through the International Physical Activity Questionnaire, 6-minute walk tests, sit-to-stand tests and handgrip strength (using Jamar Hydraulic Hand Dynamometer).

Health-related quality of life was assessed using European Quality of Life 5 Dimensions 3-Level Version and the Impact of Weight on Quality of Life-Lite questionnaires.^16^ Symptoms of depression were recorded using the Beck depression inventory II.^17^

Safety assessments included adverse event evaluation, physical examination, vital signs (blood pressure and pulse), laboratory parameters (kidney function and liver function), and pregnancy tests. An end of trial phone call was done to check for adverse events at week 28.

### Statistical Analysis

Using 20-week data from the Randomized, Controlled Trial of 3.0 mg of Liraglutide in Weight Management (SCALE) trial,^18^ 52 patients were needed to detect a difference of 5% weight loss using a 2-sample *t* test with 90% power, assuming a common SD of 5.4% for the intervention (liraglutide + lifestyle) and control groups (placebo + lifestyle), with 5% statistical significance. The sample size was increased to 66 patients (33 per group) to allow for a 20% dropout rate, and the recruitment target was set to 70 participants. Stata version 15 (StataCorp) was used to perform the sample size calculation.

The statistical analysis plan was finalized prior to database lock (February 5, 2021). The primary analyses estimated the difference in mean percentage body weight change between patients randomized to liraglutide vs placebo using a linear regression model adjusting for baseline weight, type of surgery, and diabetes status. This analysis was carried out by comparing the intervention and control groups as randomized using all available data (intention-to-treat). Additionally, a per-protocol analysis was carried out for the primary outcome as part of the secondary analyses. Linear and logistic regression models adjusting for baseline values of the outcomes where available and type of surgery and diabetes status were used to analyze the secondary outcomes. Random-effects models were used to analyze the repeated measures of the primary and secondary outcomes as part of the secondary analyses. The normality assumption was checked for each model using residual plots. If violated, a suitable transformation or nonparametric method was considered. All secondary analyses were carried out on an intention-to-treat basis. A sensitivity analysis was also carried out by replacing the missing clinic weight values by the self-reported ones for the primary outcome. A significance level of .05 was used for all hypothesis testing. *P* values were only reported for the primary analysis. Estimates of the intervention effect for each outcome are reported with 95% confidence intervals. The full statistical analysis plan can be found in Supplement 1.

## Results

**Figure 1.**
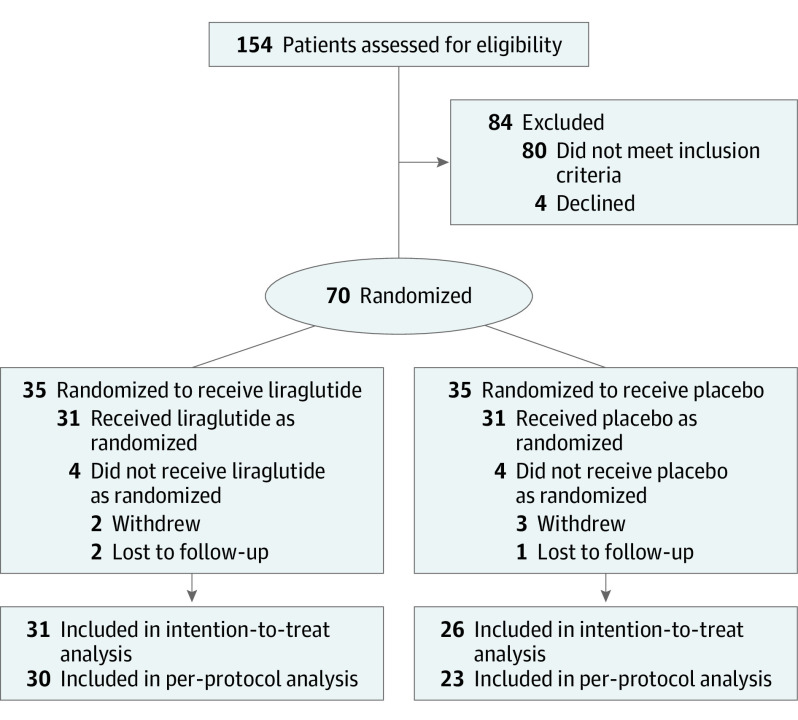
CONSORT Diagram Because of lockdown measures due to COVID-19, some participants were unable to attend the clinic for their final weight measurements. One participant in the liraglutide group and 7 in the placebo group self-reported their final body weight.

The study was conducted between September 14, 2018, and June 12, 2020. A total of 154 participants were screened for eligibility and 70 (mean [SD] age, 47.6 [10.7] years; 52 [74%] female and 18 [26%] male) were randomized: 35 to liraglutide, 3.0 mg, once daily plus lifestyle intervention and 35 to placebo plus lifestyle intervention (Figure 1). The baseline characteristics of the trial population are presented in Table 1 and eTable 3 in Supplement 2 and were comparable between groups.

**Table 1.** Baseline Characteristics

Characteristic	Mean (SD)		
	Placebo (n = 35)	Liraglutide, 3.0 mg (n = 35)	Overall trial (N = 70)
Age, y	48.4 (10.6)	46.7 (10.8)	47.6 (10.7)
Sex, No. (%)			
Female	26 (74)	26 (74)	52 (74)
Male	9 (26)	9 (26)	18 (26)
Diabetes status, No. (%)			
Type 2 diabetes	4 (11)	5 (14)	9 (13)
No diabetes	31 (89)	30 (86)	61 (87)
Metabolic surgical procedure, No. (%)			
RYGB	3 (9)	2 (6)	5 (7)
SG	32 (91)	33 (94)	65 (93)
Duration since surgery, mo	49.1 (33.7)	55.1 (33.3)	52.1 (33.4)
Percentage weight loss since surgery	7.4 (7.4)	7.0 (7.8)	7.2 (7.6)
Body mass index^a^	44.6 (8.3)	41.6 (6.9)	43 (7.5)
Body mass index,^a^ No. (%)			
<30	0	0	0
≥30 to <35	3 (7)	4 (11)	7 (10)
≥35 to <40	10 (29)	13 (33)	23 (33)
≥40	22 (62)	18 (51)	40 (57)
Race and ethnicity,^b^ No. (%)			
Asian	1 (3)	4 (11)	5 (7)
Black	9 (26)	5 (14)	14 (20)
White	22 (63)	22 (63)	44 (63)
Asian and White	0	1 (3)	1 (1)
Black Caribbean and White	1 (3)	2 (6)	3 (4)
Other multiple races or ethnicities^c^	2 (6)	1 (3)	3 (4)
Weight, kg	123.5 (24.8)	116.1 (23.6)	119.8 (24.3)
Fat mass, kg^d^	54.2 (15.1)	49.4 (11.3)	51.9 (13.5)
Lean mass, kg^d^	67.1 (13.1)	63.7 (11.0)	65.5 (12.2)
Bone density, g/cm^2^^d^	1.2 (0.1)	1.2 (0.1)	1.2 (0.1)
Fasting glucose, mmol/L	5.3 (1.5)	5.0 (1.3)	5.2 (1.4)
HbA_1c_, %	6.0 (0.9)	5.8 (0.7)	5.9 (0.8)
Heart rate, beats/min	77.3 (11.5)	74.0 (13.6)	75.7 (12.6)
Systolic BP, mm Hg	131.3 (14.5)	131.3 (15.0)	131.3 (14.7)
Diastolic BP, mm Hg	76.2 (11.2)	75.9 (10.4)	76 (10.7)
CRP, mg/L	5.3 (4.9)	6.3 (6.9)	5.9 (6)
Cholesterol, mmol/L	4.7 (0.9)	5.3 (1.2)	5 (1.1)
LDL, mmol/L	2.6 (0.9)	3.3 (0.9)	2.9 (1)
HDL, mmol/L	1.5 (0.4)	1.4 (0.4)	1.5 (0.4)
Triglyceride, mmol/L	1.4 (0.9)	1.4 (0.8)	1.4 (0.8)
Active GLP-1, pmol/L			
0 min	8.3 (8.9)	7.2 (4.4)	7.8 (7.0)
30 min	13.7 (9.8)	12.5 (6.2)	13.1 (7.2)

Abbreviations: BP, blood pressure; CRP, C-reactive protein; GLP-1, glucagon-like peptide-1; HbA_1c_, hemoglobin A_1c_; HDL, high-density lipoprotein; LDL, low-density lipoprotein; RYGB, Roux-en-Y gastric bypass; SG, sleeve gastrectomy.

^a^ Calculated as weight in kilograms divided by height in meters squared.

^b^ Race and ethnicity data were collected via patient self-report and reported to accurately describe the study population.

^c^ Other race and ethnicity groups included African, Asian British, Bangladeshi, Caribbean, Chinese, Indian, Pakistani, White British, White Irish, multiple races or ethnicities, and others, consolidated owing to small numbers.

^d^ Fat mass, lean mass, and bone mass were recorded using dual energy X-ray absorptiometry; 2 values are missing from the liraglutide group.

Three participants (4.3%), 1 (2.9%) in the placebo group, and 2 (5.7%) in the liraglutide group, were lost to follow-up. In the placebo group, 5 participants discontinued treatment but all continued to provide data. Two participants from the liraglutide group discontinued treatment, and 1 continued to provide data. All participants who completed the trial escalated to 3.0 mg once daily. Due to lockdown restrictions following the emergence of the coronavirus disease 2019 pandemic, 7 participants were unable to attend the clinic for their final body weight measurements. Additionally, 2 participants could not attend due to other health reasons, resulting in 31 participants in the liraglutide, 3.0 mg, group in the primary intention-to-treat analysis and 26 in the placebo group. Lockdown measures impacted upon conduct of final visit blood tests, dual energy X-ray absorptiometry, and physical functional testing.

**Figure 2.**
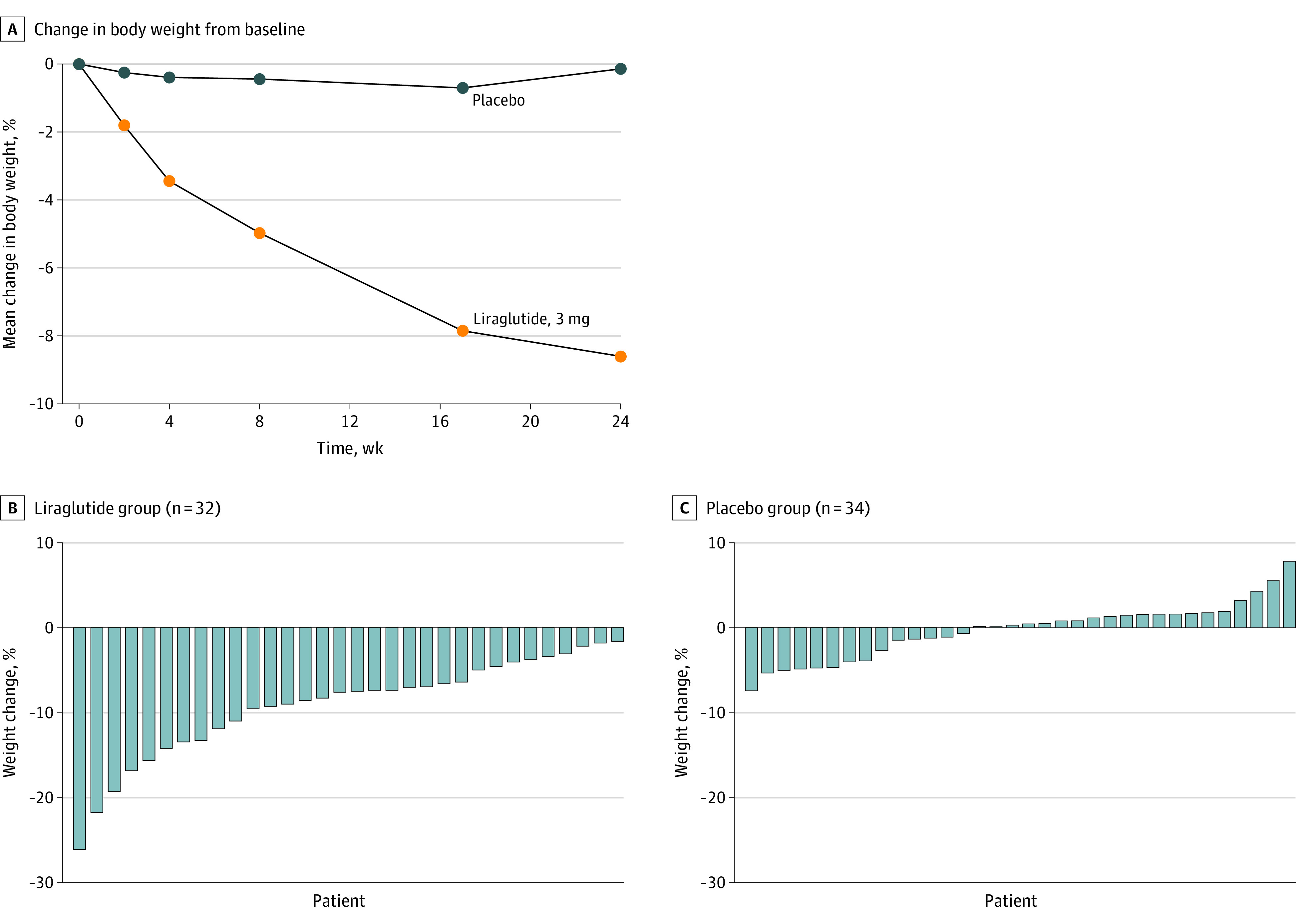
Effect of Liraglutide, 3.0 mg, Once Daily vs Placebo Over Time

From baseline to week 24, a greater reduction in percentage body weight was observed in the 3.0-mg liraglutide group compared with the placebo group (mean [SD], −8.82 [4.94] vs −0.54 [3.32], respectively; *P* < .001) (Figure 2). The mean difference in percentage body weight change was −8.03 (95% CI, −10.39 to −5.66) (eFigure 2 and eTable 1 in Supplement 2).

A per-protocol analysis for those with an in-person body weight measurement (30 participants in the liraglutide, 3.0 mg, group and 23 participants in the placebo group) showed greater percentage change in body weight in the liraglutide, 3.0 mg, group compared to the placebo group (mean [SD], −9.05 [4.85] vs −0.86 [3.23], respectively) with an adjusted mean difference of −7.67 (95% CI, −10.14 to −5.21; *P* < .001); this is consistent with the results for the primary analysis.

Self-reported weights were used in addition to the original data available for a sensitivity analysis, resulting in 32 participants in the liraglutide, 3.0 mg, group and 34 in the placebo group. The mean (SD) percentage change in body weight from baseline to 24 weeks was −8.65 (4.96) in the liraglutide, 3.0 mg, and −0.14 (3.28) in the placebo group with an adjusted mean difference of −8.29 (95% CI, −10.42 to −6.16).

Next, we examined the effect of liraglutide, 3.0 mg, compared to placebo on categorical weight loss (eTable 2 and eFigure 3 in Supplement 2). A greater proportion of participants in the liraglutide group compared to the placebo group lost 5% or more of their body weight (71.9% vs 8.8%). Waterfall plots (Figure 2) illustrate the range of weight loss responses in the liraglutide and placebo groups, respectively.

Results from the anthropometric, cardiometabolic, and physical function secondary outcome analyses are presented in Table 2 and eTables 4-6 in Supplement 2. Total mean (SD) body weight reduction from baseline to end of treatment was greater in the liraglutide, 3.0 mg, group compared to the placebo group (−9.5 [5.1] kg vs −0.4 [3.9] kg, respectively) with an adjusted treatment difference of −9.2 kg (95% CI, −11.5 to −6.9). Total body fat decrease from baseline to week 24 was greater in the liraglutide group compared to the placebo group (adjusted mean difference, −4.9 kg; 95% CI, −7.2 to −2.5). Favorable changes in fasted glucose, hemoglobin A_1c_, systolic blood pressure, total cholesterol, and high-density lipoprotein cholesterol were observed in the liraglutide, 3.0 mg, group compared to the placebo group (Table 2).

**Table 2.** Multiple Linear Regression Analyses of the Change in Secondary Outcomes

Outcome	Placebo		Liraglutide		Treatment effect (adjusted mean difference)^b^
	No.	Mean (SD)^a^	No.	Mean (SD)^a^	
Weight loss, kg	34	−0.39 (3.88)	32	−9.49 (5.07)	−9.16 (−11.45 to −6.87)
Fat mass, kg	24	0.68 (3.91)	23	−4.10 (4.23)	−4.85 (−7.18 to −2.53)
Lean mass, kg	24	−1.15 (3.25)	23	−4.16 (3.00)	−3.22 (−4.80 to −1.64)
Bone density, g/m^2^	24	0.01 (0.04)	23	−0.01 (0.02)	−0.00 (−0.02 to 0.02)
Glucose, mmol/L	27	−0.02 (0.88)	27	−0.43 (0.81)	−0.51 (−0.86 to −0.17)
HbA_1c_, %	27	−0.03 (0.22)	26	−0.27 (0.37)	−0.24 (−0.32 to −0.16)
Heart rate, beats/min	31	2.35 (11.55)	30	4.57 (12.38)	0.71 (−4.75 to 6.16)
Systolic BP, mm Hg	32	2.34 (18.93)	32	−6.28 (16.40)	−9.05 (−16.24 to −1.85)
Diastolic BP, mm Hg	32	−0.34 (13.22)	32	−0.38 (13.20)	−0.34 (−5.81 to 5.14)
CRP, mg/L	26	−0.49 (2.82)	27	−1.22 (2.56)	−0.88 (−2.09 to 0.32)
Cholesterol, mmol/L	31	0.10 (0.48)	28	−0.47 (0.58)	−0.42 (−0.68 to −0.15)
LDL, mmol/L	30	−0.03 (0.32)	28	−0.26 (0.58)	−0.05 (−0.29 to 0.20)
HDL, mmol/L	31	0.02 (0.21)	28	−0.10 (0.18)	−0.12 (−0.23 to −0.01)
Triglyceride	31	0.21 (1.55)	28	−0.22 (0.66)	−0.40 (−1.04 to 0.24)
BDI-II score	33	−1.91 (9.43)	32	−5.66 (8.59)	−3.23 (−6.99 to 0.53)
IWQOL-Lite scores					
Total	33	−0.93 (10.70)	32	4.98 (15.05)	7.13 (0.60 to 13.66)
Physical function	33	−0.28 (11.29)	32	5.54 (18.48)	7.54 (0.10 to 14.99)
Self-esteem	33	−3.57 (15.72)	32	4.60 (21.30)	8.77 (−0.51 to 18.06)
Sex life	32	−2.93 (20.82)	31	4.64 (28.27)	7.00 (−5.60 to 19.60)
Public distress	33	1.97 (18.45)	32	5.31 (16.80)	5.04 (−3.89 to 13.98)
Work	33	−1.52 (17.19)	31	1.21 (21.07)	3.12 (−6.43 to 12.66)

Abbreviations: BP, blood pressure; BDI, Beck depression inventory; CRP, C-reactive protein; HbA_1c_, hemoglobin A_1c_; HDL, high-density lipoprotein; IWQOL, Impact of Weight on Quality of Life; LDL, low-density lipoprotein.

^a^ Estimated mean difference (SD) from baseline.

^b^ Estimated from linear regression model adjusting for baseline value of the secondary outcome, baseline weight, type of surgery, and diabetes status.

Reported adverse events and their frequency for the liraglutide and placebo groups are illustrated in Table 3. Adverse events, predominantly gastrointestinal, were more frequent with liraglutide, 3.0 mg (28 events [80%]), than placebo (20 events [57%]). There were no serious adverse events in either group, no reports of acute cholecystitis or pancreatitis, and no treatment-related deaths.

**Table 3.** Adverse Events (AE)^a^ in the BARI-OPTIMISE Study Population

Event	Participants who experienced an AE, No. (%)		
	Placebo (n = 35)	Liraglutide (n = 35)	Total (N = 70)
Total	20 (57)	28 (80)	48 (67)
Total AEs, No.^b^	75	37	112
Gastrointestinal events			
Nausea	7 (20)	18 (51)	25 (36)
Diarrhea	2 (6)	2 (6)	4 (6)
Constipation	2 (6)	9 (26)	11 (16)
Vomiting	1 (3)	1 (3)	2 (3)
Abdominal pain	1 (3)	2 (6)	3 (4)
Abdominal bloating	0	1 (3)	1 (1)
Dyspepsia	0	1 (3)	1 (1)
General and administration site events			
Headache	2 (6)	1 (3)	3 (4)
Injection site reaction	3 (9)	2 (6)	5 (7)
Urticaria	0	1 (3)	1 (1)
Fatigue	2 (6)	5 (14)	7 (10)
Insomnia	2 (6)	0	2 (3)
Cardiovascular events			
Dizziness	2 (6)	3 (9)	5 (7)
Palpitations	1 (3)	3 (9)	4 (6)
Infections			
Upper respiratory tract infection	2 (6)	5 (14)	7 (10)
Influenza	2 (6)	3 (9)	5 (7)
Metabolic and nutritional events			
Decreased appetite	3 (9)	11 (31)	14 (20)
Dry mouth	2 (6)	3 (9)	5 (7)
Musculoskeletal events			
Back pain	0	1 (3)	1 (1)
Arthralgia	2 (6)	2 (6)	4 (6)
Serious AEs	0	0	0

^a^ Grouped by system organ class as per the Medical Dictionary for Regulatory Activities (http://www.meddra.org). Events were included if they occurred on or after the first dose of study drug was administered and until the end of the trial, 4 weeks after the last day the last dose of study drug was administered.

^b^ Some participants experienced multiple AEs.

## Discussion

Although metabolic surgery remains the most effective and durable therapy for severe obesity and associated comorbidities, 1 in 4 patients experience poor weight loss outcomes. Thus, treatment of these patients remains a clinical challenge with a paucity of randomized clinical trials.

To our knowledge, the BARI-OPTIMISE trial is the first randomized clinical trial to evaluate the efficacy and safety of liraglutide, 3.0 mg, compared to placebo as an adjunct to a lifestyle intervention in people with suboptimal weight loss after metabolic surgery. Our findings show that liraglutide, 3.0 mg, for 24 weeks led to a significantly greater reduction in percentage body weight compared to placebo, coupled with reduced fat mass, favorable changes in cardiometabolic risk factors, and improvement in health-related quality of life. At the end of the 24-week treatment period, 71.9% of participants treated with liraglutide, 3.0 mg, compared with 8.8% in the placebo group lost 5% or more of their baseline body weight, a widely used criterion to determine a clinically meaningful response.

The estimated treatment difference of −8.03% (95% CI, −10.39 to −5.66) compared to placebo reflects greater weight loss compared to corresponding trials of liraglutide, 3.0 mg, in people with overweight or obesity who have not undergone metabolic surgery.^18^ A recent systematic review and meta-analysis of randomized clinical trials (n = 6028) evaluating the efficacy of liraglutide, 3.0 mg, in adults with overweight or obesity treated for at least 1 year reported a mean difference in body weight change of −4.8% (95% CI, −5.6 to −4.1) relative to placebo.^19^ Importantly, participants in our trial did not reach weight loss nadir at the end of the 24-week treatment period, suggesting further weight reduction and health benefits may be achievable with a longer treatment period.

In the BARI-OPTIMISE trial, a mean (SD) percentage weight change of −0.54 (3.32) was seen in the placebo group. This represents comparatively lower weight loss than placebo groups receiving lifestyle interventions in weight management trials.^18,19^ This is likely due to the fact that the post–metabolic surgery patient population, unlike participants recruited to weight management trials who are often treatment naive, have already spent many years in weight management programs with lifestyle modification. The lack of weight loss through energy deficit in our placebo group may also reflect biological drivers for poor weight loss and highlight the need for adjuvant therapies.

Compared to trials of liraglutide, 3.0 mg, for people with overweight or obesity who have not undergone bariatric surgery, patients in our cohort reported fewer gastrointestinal events, and all participants were able to escalate to the full 3.0-mg dose, suggesting liraglutide, 3.0 mg, was better tolerated in this patient population.^20,21^

Our results show greater absolute weight loss, with an adjusted treatment effect difference of −9.2 kg (95% CI, −11.5 to −6.9), compared to the GRAVITAS randomized clinical trial^13^ where liraglutide, 1.8 mg, once daily was evaluated as an adjunct for the treatment of persistent or recurrent type 2 diabetes following RYGB or SG. In addition, we reported a greater number of participants who lost 5% or more of their baseline body weight treated with liraglutide (71.9%) compared to the GRAVITAS trial (46%). These differences may be due to differences in the study populations, the greater efficacy of the 3.0-mg dose on weight reduction than the 1.8-mg dose,^22^ or our approach of selecting participants with a suboptimal nutrient-stimulated GLP-1 response.

### Strengths and Limitations

The BARI-OPTIMISE trial has several strengths. Prior to recruitment, patients had undergone a multidisciplinary assessment for additional contributors to poor weight loss. The study was randomized, placebo-controlled, and double-blinded and included comprehensive assessment of body composition, cardiometabolic risk factors, physical function, and health-related quality of life.

Our study also had limitations. We only recruited patients following primary surgery; however, a recent retrospective study reported that liraglutide, 3.0 mg, was equally effective for management of poor weight loss after primary or revisional metabolic surgery.^23^ We recruited people with 20% or less postsurgical weight loss, which is 1 of the accepted criteria used to define suboptimal weight loss, despite the lack of a formal definition. Additional limitations include that most participants were White and female, which is representative of the UK bariatric surgery population but not the global population of people with obesity. The clinical effectiveness of liraglutide, 3.0 mg, was only assessed in patients with a suboptimal GLP-1 response and not compared in those with optimal GLP-1 responses. Therefore, conclusions surrounding the indications for this targeted approach cannot be drawn. Studies investigating the relationship between postsurgery meal-stimulated GLP-1 profiles and response to GLP-1 receptor agonists are required. Due to COVID-19 restrictions, 7 final in-person body weight assessments were not taken. Furthermore, due to participants who were lost to follow-up, discontinued treatment, or were unable to attend their final visit, the number included in the intention-to-treat analysis had to be reduced to 31 in the liraglutide group and 26 in the placebo group. However, this did not affect the power to detect a difference in treatment effect, which was both statistically and clinically significant. Additionally, during the 24-week treatment period, weight loss did not plateau, suggesting a longer treatment period may be necessary to achieve maximal benefits of liraglutide, 3.0 mg, in this patient population.

Newer gut hormone–based therapies with greater efficacy than liraglutide, 3.0 mg, are emerging.^21,24,25^ Randomized clinical trials investigating the efficacy of novel pharmaceutical agents will be needed to generate the evidence required to deliver individualized precision-medicine approaches to patients with obesity and suboptimal weight loss following metabolic surgery.

## Conclusions

In conclusion, 24 weeks of liraglutide, 3.0 mg, as an adjunct to a lifestyle intervention in people with poor weight loss and a suboptimal GLP-1 response after metabolic surgery, was safe and well tolerated and led to clinically meaningful reductions in body weight. Our findings therefore suggest that liraglutide, 3.0 mg, may have a role in the treatment of people with poor weight loss following metabolic surgery.

## Notes

Supplement 1. Trial protocol and statistical analysis plan

Supplement 2. eTable 1. Analysis of primary outcome at multiple time points eTable 2. Patients that lost ≥ 5%, ≥ 10% and ≥ 15% of their body weight at 24 weeks eTable 3. Baseline values for functional capacity tests, BDI score, IWQOL-Lite score, IPAQ activity and EQ-53-3L score eTable 4. Analysis of physical function tests between baseline and week 24 eTable 5. Analysis of IPAQ questionnaire between baseline and week 24 eTable 6. Analysis of IQWOL-Lite and components between baseline and week 24 eFigure 1. Trial design eFigure 2. Analysis of change in weight at multiple time points (kg) eFigure 3. Effect of liraglutide 3.0mg once daily and placebo on categorical weight loss

Supplement 3. Data sharing statement
