# Supplementary material for: Safety and Efficacy of Liraglutide, 3.0 mg, Once Daily vs Placebo in Patients With Poor Weight Loss Following Metabolic Surgery: The BARI-OPTIMISE Randomized Clinical Trial
Source: JAMA Surg. 2023 Jul 26;158(10):1003–11. doi: 10.1001/jamasurg.2023.2930 (PMC10372755; doi:10.1001/jamasurg.2023.2930)
Supplement: Supplement 1. — Trial protocol and statistical analysis plan [file jamasurg-e232930-s001.pdf]

Study protocol

**Full title of trial**	A double-blinded, randomised, placebo-controlled trial of liraglutide 3.0 mg in patients with poor weight-loss and a suboptimal glucagon-like peptide-1 response following bariatric surgery.
**Short title**	BARI-OPTIMISE
**Version and date of protocol**	Version 4, 17.01.2019
**Sponsor:**	University College London (UCL)
**Sponsor protocol number**	17/0238
**Funder (s):**	National Institute for Health Research (NIHR)<br>The Sir Jules Thorn Charitable Trust
**Clinicaltrials.gov no:**	NCT03341429
**Universal Clinical Trial Number:**	U1111-1185-8283
**EudraCT Number:**	2017-002407-10
**Phase of trial:**	Phase IV

Background

Obesity and its associated co-morbidities represent a global health threat causing 3.4 million preventable deaths annually. Bariatric surgery is the most effective treatment for patients with severe obesity producing sustained weight-loss with reduced morbidity and mortality. As a consequence of its unparalleled health benefits, bariatric surgery has been widely adopted with ~500,000 operations undertaken annually world-wide [1]. In the UK, patients with severe obesity, defined as a body mass index (BMI) of ≥40 kg/m^2, or ≥35 kg/m^2 with an obesity-associated co-morbidity, are eligible for bariatric surgery in accordance with National Institute for Health and Clinical Excellence (NICE) Guidelines [2].

Variability in weight-loss response following bariatric surgery

Roux-en-Y gastric bypass (RYGB) and sleeve gastrectomy (SG) are the commonest bariatric procedures performed globally, accounting for 42% and 37% of operations undertaken in 2013 respectively [1]. Whilst at a population level these operations are highly effective at reducing weight it is now clear that, at the level of the individual, weight-loss following RYGB and SG is highly variable [3, 4]. Figure 1 shows the variability in 2-year percentage weight-loss (%WL) following RYGB and SG from our unit. Given the associated surgical risks, procedure cost and the need for lifelong nutritional monitoring there is an urgent unmet clinical need to improve weight-loss following bariatric surgery and improve the health of bariatric surgery patients.

Figure 1: Weight-loss in patients who undergo sleeve gastrectomy or Roux-en-Y gastric bypass is highly variable

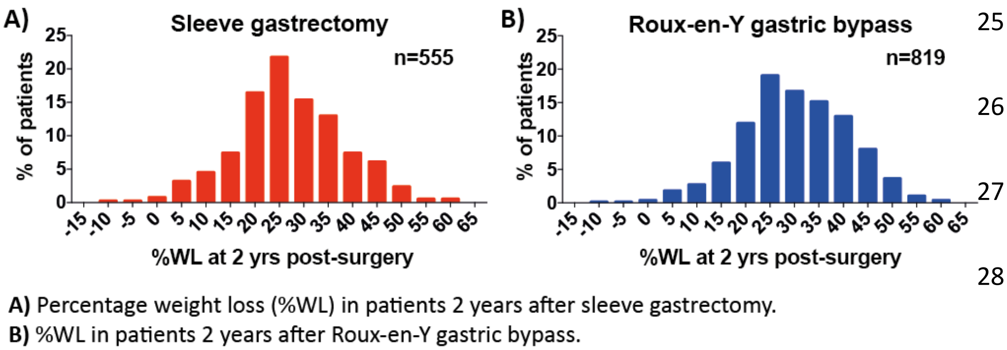

A) Percentage weight loss (%WL) in patients 2 years after sleeve gastrectomy.
B) %WL in patients 2 years after Roux-en-Y gastric bypass.

The importance of maximising post-surgery weight-loss

Following RYGB and SG, glycaemic control improves rapidly by weight-loss independent mechanisms [5]. However, there is increasing evidence that long-term T2D remission depends on weight-loss [6-8]. In our patients, complete T2D remission rates at 2-years post-surgery (defined as HbA1c <6%, off all medication for more than 12 months) are determined by %WL, independent of procedure type (Figure 2). Importantly, multivariate logistic regression analysis correcting for confounding baseline factors shows that the odds of complete T2D remission increase by 10% for every additional 1%WL. Therefore, our data highlight the important clinical impact that small changes in %WL have on T2D remission, which has been shown to lead to a reduction in long-term microvascular disease outcomes [9]. Furthermore, the resolution and/or improvement of hypertension, dyslipidaemia, obstructive sleep apnoea and non-alcoholic steatohepatitis following bariatric surgery are positively associated with %WL [10-13] with weight regain leading to relapse of these co-morbidities [10, 11]. In addition, greater improvements in health-related quality-of-life (HRQoL) outcomes following bariatric surgery are reported with greater weight-loss [14, 15]. Taken together these findings represent a strong rationale for maximising weight-loss following bariatric surgery.

Figure 2: Rate of remission of type 2 diabetes (T2D) following sleeve gastrectomy or Roux-en-Y gastric bypass is determined by weight-loss independent of procedure type

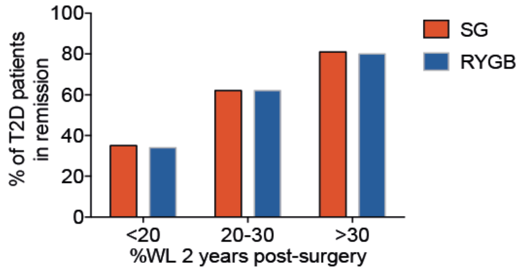

Percentage of patients with T2D at the time of surgery in complete remission at 2 years after sleeve gastrectomy (n=118) or Roux-en-Y gastric bypass (n=116) plotted according to percent weight-loss category (<20%, 20-30% and >30%).

Rationale

Gut hormones levels in patients with poor weight-loss/ weight regain

The gastrointestinal tract is the body's largest endocrine organ, secreting a panoply of gut peptides in response to nutrient ingestion that play a key role in regulating energy and glucose homeostasis. Decreased caloric intake, due to reduced appetite, is the primary weight-loss driver following RYGB and SG in humans. The mechanisms underlying reduced post-surgery appetite remain to be fully elucidated, but post-operative changes in circulating gut hormones, in particular peptide YY (PYY), ghrelin and glucagon-like-peptide-1 (GLP-1) are key contenders [16, 17]. Indeed, cross-sectional studies show that patients with poor weight-loss post-RYGB exhibit increased appetite and caloric intake with higher acyl-ghrelin levels and lower GLP-1 and PYY circulating levels compared to patients with a good weight-loss response [18, 19].

The GLP-1 analogue liraglutide 3.0 mg reduces body weight and adiposity when administered daily subcutaneously to obese patients [20-24]. Importantly, a retrospective analysis of the effect of short-term (average duration 12 weeks) use of the synthetic GLP-1 analogue liraglutide in 15 patients with poor weight-loss following bariatric surgery found a significant reduction in %WL [21].

Our unpublished data show that patients with a poor weight-loss response (<20%) following SG and RYGB exhibit an attenuated post-surgery gut hormone response compared to patients with a good weight-loss response (>20%). In particular, we found that a proportion of patients with poor weight-loss exhibited a lower nutrient-stimulated GLP-1 response (delta active GLP-1 time 0 to time 30 minutes and active GLP-1 area under-the-curve) compared to patients with good weight-loss (Figure 3). This finding suggests that a pharmaceutical approach tailored to the patient's post-surgery gut hormone profile would improve %WL. Based upon these studies, an increment in active GLP-1 of less than 2-fold in the first 30 minutes following our standard meal test was identified as strongest predictor of poor %WL.

Figure 3: Patients with poor weight-loss following RYGB and SG have an attenuated gut hormone response compared to patients with a good weight-loss response

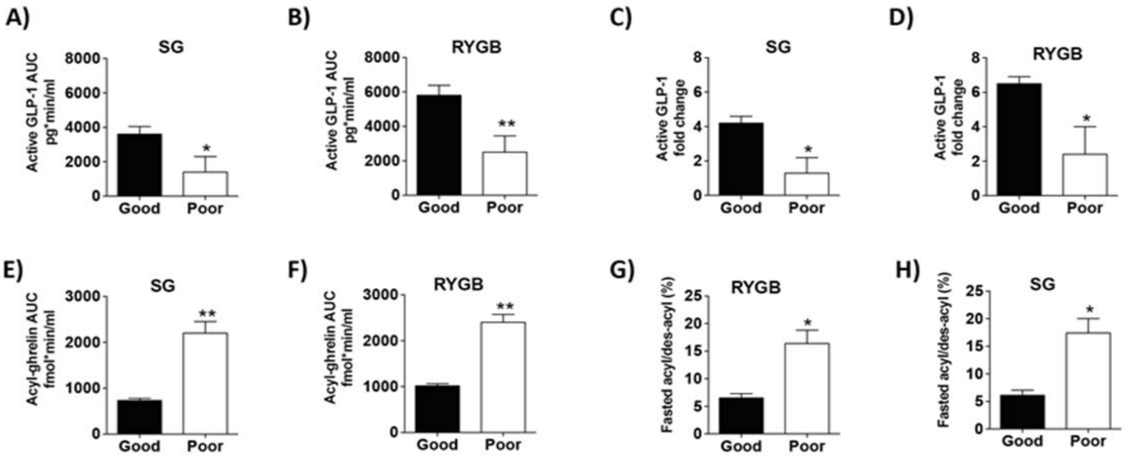

Patients with a poor weight-loss response (< 20%WL) following SG (n=10) and RYGB (n=10) and good response (%WL>20%) following SG (n=10) and RYGB (n=10) attended after an overnight fast for a 500 kcal test meal at time zero. Blood samples were collected at 15, 30, 60, 90, 120, 150 and 180 minutes post-meal. Active GLP-1 AUC in **(A)** SG patients and **(B)** RYGB patients and active GLP-1 fold-change from baseline at 30 minute post-meal in **(C)** SG and **(D)** RYGB were calculated for good and poor weight-loss responders. Acyl-ghrelin AUC in **(E)** SG and **(F)** RYGB and the fasted acyl/desacyl-ghrelin ratio in **(G)** RYGB and **(H)** SG patients were calculated in good and poor responders. Data are mean ± sem. * p<0.05, **p<0.01, ***p<0.001.

Hypothesis

Currently, there are no available pharmacological treatments for patients with poor weight loss after surgery and the only therapeutic option is revision surgery, which carries a mortality risk and is difficult to access. Based upon our data, we hypothesise that GLP-1 analogue administration to patients with poor weight-loss and a suboptimal GLP-1 response following RYGB or SG will lead to a greater reduction in body weight, adiposity and improvement in health and HRQoL outcomes compared to placebo.

Objectives

To determine whether 24 weeks of subcutaneous liraglutide 3.0 mg causes greater %WL, reduction in adiposity and improvement in metabolic indices, physical function and HRQoL than placebo in patients with suboptimal nutrient-stimulated GLP-1 response and poor weight-loss following RYGB or SG.

Primary objective

The primary objective of this trial is to compare the efficacy of 24 weeks of subcutaneous liraglutide 3.0 mg versus placebo administration, as an adjunct to diet and exercise, on %WL in participants with poor weight-loss and a sub-optimal active GLP-1 response following primary RYGB or SG at the end of the 24-week treatment period.

Secondary objectives

To compare the effect of 24 weeks of subcutaneous liraglutide 3.0 mg versus placebo administration as an adjunct to diet and exercise in participants with poor weight-loss and a sub-optimal active GLP-1 response following primary RYGB or SG, at the end of the 24-week treatment period, upon:

1. Change in fat, lean body mass and bone density.
2. Change in circulating fasted glucose, insulin, HbA1c and leptin, and meal-stimulated glycaemic index, gut hormones and appetite response.
3. Change in HRQoL measures.
4. Change in physical functional assessments and activity levels.
5. Change in healthcare service usage.

Trial design

This study is a double-blind, randomised, placebo-controlled, two-arm, parallel group, single-site trial (Figure 4). The purpose of this trial is to evaluate the therapeutic effects of liraglutide 3.0 mg in patients with 'poor' weight loss and a suboptimal glucagon-like peptide-1 response following bariatric surgery. The trial will test whether a 24-week administration of liraglutide 3.0 mg can improve weight loss in patients 1 year or more since primary RYGB or primary SG. Seventy participants will be enrolled to receive liraglutide 3.0 mg (n = 35) or placebo (n = 35) for 24 weeks. An identical placebo containing no active ingredients will be used as a comparator to evaluate the real treatment effect. Treatment allocation will be concealed from patients and investigators. A dose of 3.0 mg that is approved for weight loss will be used. This trial will test a longer treatment duration of 24 weeks as to extend the findings from a previous study that evaluated a treatment period of only 12 weeks [21]. Subject visits will be carried out at weeks 2, 4, 8, 17 and 24 of the treatment initiation. End-of-study visit will be over the phone 4 weeks after the end of treatment (i.e. week 28).

Figure 4: Flow chart of the BARI-OPTIMISE Trial

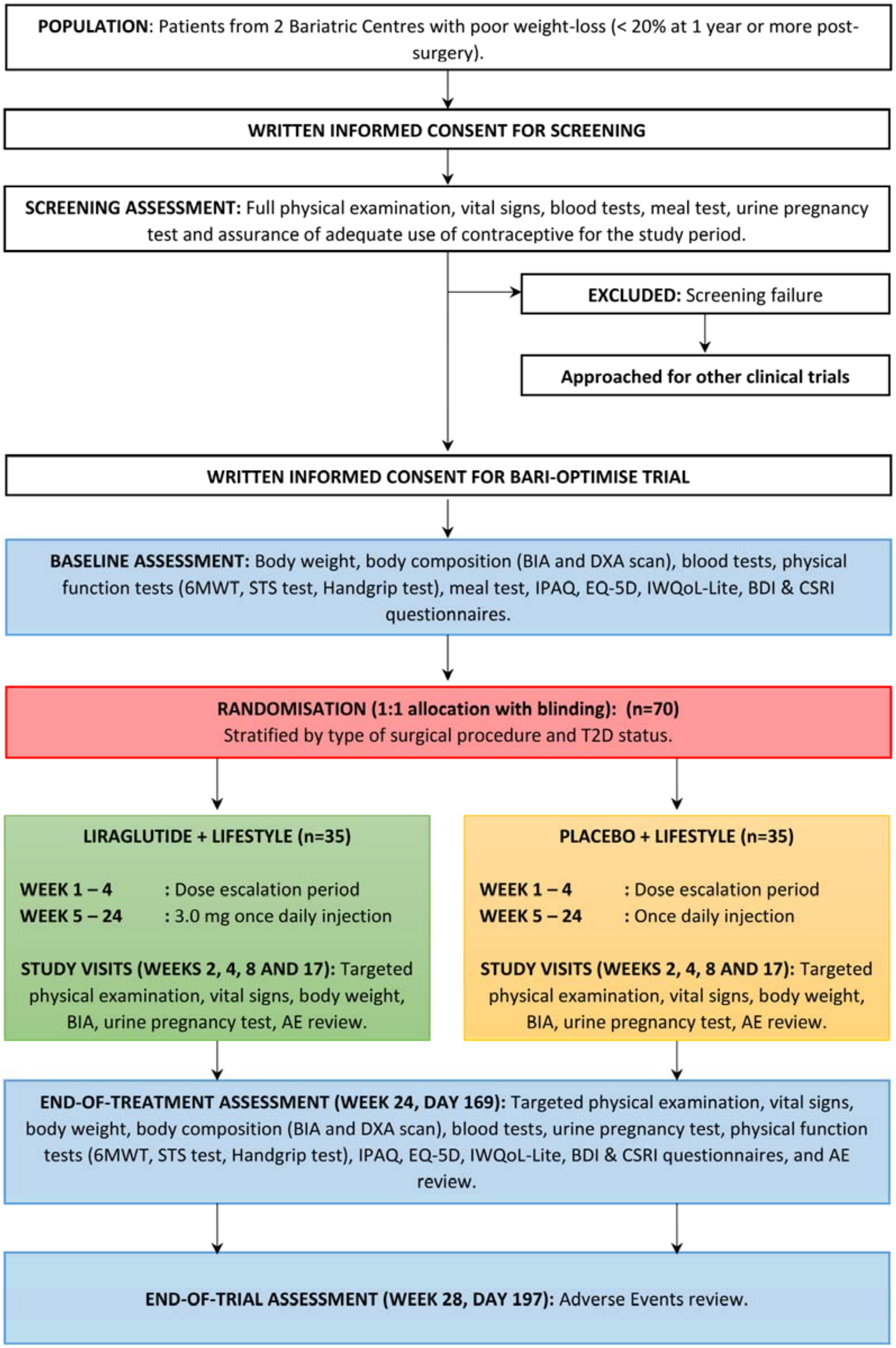

Abbreviation: BDI: Beck Depression Inventory, BIA: Bioelectrical Impedance Analysis, CSRI: Client Service Receipt Inventory (adapted), DXA: Dual Energy X-Ray Absorptiometry, EQ-5D: EuroQol-5D, HRQoL: Health-

Related Quality of Life, IWQoL-Lite: Impact of Weight on Quality of Life-Lite, PA: Physical Activity, STS: Sit-to-stand, T2D: Type 2 Diabetes, 6MWT: 6-Minute Walk Test.

Selection of Subjects

Study population: patients, 1 year or more following primary RYGB or primary SG, with poor weight-loss response (<20% WL) will be invited to participate. There will be no waivers to the inclusion and exclusion criteria.

Inclusion criteria

1. Patients, 1 year or more since primary RYGB or primary SG, with poor weight-loss (<20% WL) that is not caused by either a surgical or psychological problem.
2. Adults, 18-64 years inclusive.
3. Suboptimal nutrient-stimulated GLP-1 response assessed by a meal test. Suboptimal active GLP-1 response is defined as a ≤2-fold increase in active GLP-1 circulating levels between time 0 and time 30 minutes.
4. Females of childbearing potential and males must be willing to use highly effective method of contraception (Appendix 2) from the time consent is signed until 6 weeks after treatment discontinuation.
5. Females of childbearing potential must have a negative pregnancy test within 7 days prior to being registered for trial treatment. NOTE: Subjects are considered not of child bearing potential if they are surgically sterile (i.e. they have undergone a hysterectomy, bilateral tubal ligation, or bilateral oophorectomy) or they are postmenopausal.
6. ≤5 % variation in body weight over preceding 3 months.
7. Fluent in English and able to understand and complete questionnaires.
8. Willing and able to provide written informed consent and comply with the trial protocol.

Exclusion criteria

1. Had a surgical procedure other than gastric bypass and sleeve gastrectomy, or revision bariatric surgery of any operation type.
2. Pregnant or lactating mothers.
3. Participation in other clinical intervention trial.
4. Lifetime history of suicidal behaviour or severe depression assessed by direct questioning.
5. Clinically significant medical abnormalities (e.g., unstable hypertension, clinically significant ECG abnormalities, liver cirrhosis, AST or ALT > 3x the upper normal limit).
6. Heart rate ≥ 100 beats/minute at screening on two separate measurements.
7. Uncontrolled hypertension (systolic blood pressure ≥ 160 mmHg or diastolic blood pressure ≥ 100 mmHg).
8. Renal impairment (estimated glomerular infiltration rate (eGFR <30 ml/min 1.73 m^2)
9. Known or suspected hypersensitivity to liraglutide 3.0 mg and placebo or any of the excipients involved in their formulation.
10. Personal or family history of medullary thyroid carcinoma or multiple endocrine neoplasia syndrome type 2.

11. Personal history of pancreatitis.
12. Uncontrolled hypothyroidism or hyperthyroidism.
13. History of stroke, unstable angina, acute coronary syndrome, congestive heart failure New York Heart Association class III-IV within the preceding 12 months.
14. History of arrhythmias.
15. Inflammatory bowel disease.
16. Diabetic gastroparesis.
17. Concomitant GLP-1 receptor agonist usage.
18. Concomitant usage of medications that cause weight gain or weight loss.
19. Concomitant usage of DPPIV-inhibitors.
20. Insulin usage.

Recruitment

Participant recruitment will only commence when the trial has been confirmed by the Sponsor, issued a Trust Confirmation of Capacity and Capability and the trial site has received the Open to Recruitment Letter. The clinical bariatric teams at UCLH, the Homerton Hospital and the Whittington Hospital specialist bariatric centres (800 patients undergoing bariatric surgery per annum) will identify patients fulfilling the eligibility criteria. The Whittington Hospital will act as PIC and the trial will be undertaken at UCLH. Patients will be considered to be enrolled into the trial following: consent, baseline assessments, randomisation, allocation of the participant trial number and intervention.

Study procedures and schedule of assessments

Informed consent procedure

The person taking consent will be GCP trained, suitably qualified and experienced, and have been delegated this duty by the CI. At first, suitable patients will be identified by the bariatric team during their post-surgery follow-up visits at bariatric centre and asked if they are interested to take part in the study. Verbal consent will be sought from interested patients before they are approached by a research investigator who will explain the screening procedure as well as the aims, methods, anticipated benefits and potential hazards of the study. The investigator will also explain that patients are under no obligation to undergo screening and enter the trial and that they can withdraw at any time without having to give a reason. Those patients interested in taking part to the trial will be asked to sign a first consent form in order to undergo screening for this trial, as the assessment includes a meal test that is considered research procedure. Written informed consent will be sought within one week with a minimum of 24 hours after being approached and given the Participants Information Sheet (PIS). An original copy of the signed informed consent form will be given to the participant in addition to the original copy that will be filed in the Trial Master File (TMF). A copy will be placed in the medical notes. In addition, the consent process will be documented in the medical notes for a clear audit trail.

If a patient does not meet all the inclusion and exclusion criteria no further action will be taken and the patient will be informed that, for their own safety, it is not appropriate they continue with the study. Screening failure patients might be contacted for other research studies should they be interested.

A research investigator will notify patients of screening outcome via phone call. The investigator will explain again study aims, methods, anticipated benefits and potential hazards to qualifying patients and answer any questions they may have. Eligible patients will be asked to sign a second consent form, the study consent form, to confirm their willingness to take part to the trial. No study procedures will be conducted prior to the patient giving consent by signing the consent form. The investigator will also explain that patients are under no obligation to enter the trial and that they can withdraw at any time during the trial, without having to give a reason. Written informed consent will be sought within one week with a minimum of 24 hours after being approached and given the study PIS.

An original copy of the signed informed consent form will be given to the participant in addition to the original copy that will be filed in the Trial Master File (TMF). A copy will be placed in the medical notes. In addition, the consent process will be documented in the medical notes for a clear audit trail.

If new safety information results in significant changes in the risk/benefit assessment, the PIS and consent form will be reviewed and updated if necessary and subjects will be re-consented as appropriate. Therefore, the version and date of the PIS and ICF in use at the time will be recorded in the medical notes.

The investigator obtaining consent will register the participant for the study by entering all baseline data regarding the participant on the database. The system will then assign a unique participant identification number (PIN) to that participant that needs to be recorded on the consent form. No eligibility waivers or deviations will be permitted.

Randomisation Procedures

Following participant screening, consent and baseline measure collection, the randomisation procedure will be remotely carried out by the Sealed Envelope, an independent specialised company that provides 24/7 cover to undertake the randomisation, and unblinding if required. The type of randomisation to be used is a stratified block randomisation with random block sizes. Subjects will be randomly assigned in a 1:1 ratio to receive either liraglutide 3.0 mg or placebo, stratified for type of surgical procedure and T2D status. This will be done by accessing the randomisation system website through the internet. The investigator will provide participant's initials, date of birth and stratification information before a randomisation code can be generated for each participant. Both participants and investigators will be blinded to study-group assignments. A randomisation notification message will be automatically generated to confirm the success of the randomisation process to the investigator, no group allocation will be disclosed. A randomisation notification email will be generated and sent to the pharmacist reporting the participant randomisation code; this randomisation code will reveal group allocation when identified in the code list. Only the trial coordinator and the trial pharmacist will have access to the code list. The blinding of the trial will be maintained throughout the trial until all data entry and processing are complete, the database has been locked and data analysis performed. Participants will be given a 24-hour Contact Card for emergency unblinding if required, medical support, or for any enquiries they have throughout the study period. For details on unblinding procedure refer to the trial Randomisation, Unblinding and Code break SOP.

Unblinding

The trial code will only be broken for valid medical or safety reasons e.g. in the case of a severe adverse event where it is necessary for the treating health care professional to know which treatment the participant is receiving before providing appropriate treatment. Subject always to clinical need, where possible, members of the research team will remain blinded. For details on unblinding procedure refer to the trial Randomisation, Unblinding and Code break SOP.

In the event a code is required to be unblinded a formal request for unblinding will be electronically submitted by the authorised investigator to Sealed Envelope using the online unblinding facility, available 24 hours daily. Alternatively, a request to the holder of the code break list, or their delegate, will be made and the unblinded information obtained. The pharmacist and the Trial Co-ordinator will be the solely holders of the code list, other than Sealed Envelope.

If a treating physician, who is not the CI/PI/trial investigator, requires the treatment to be unblinded in an emergency situation, they should notify the Investigating team that an emergency unblinding is required for a trial subject. The investigator/research team will provide this information as quickly as possible. On receipt of the treatment allocation details the CI/PI/trial investigator, or treating health care professional, will deal with the participant's medical emergency as appropriate.

The CI/PI will document the breaking of the code and the reasons for doing so on the CRF/data collection tool, in the site file and medical notes. It will also be documented at the end of the study in any final study report and/or statistical report. The CI/Investigating team will notify the JRO (acting on behalf of the Sponsor) in writing as soon as possible following the code break detailing the necessity of the code break.

The CI/PI will also notify the relevant authorities. The written information will be disseminated to the Data and Safety Monitoring Committee (DSMC) for review in accordance with the DSMC Charter.

Unblinding for the submission of SUSAR reports:

The following procedure will be used to unblind for the submission of a SUSAR report to the regulatory agencies:

- A member of the JRO Sponsor's office will contact the pharmacy requesting unblinding information from the randomisation list.
- The pharmacist will provide their email address and name for the request to be formalised in an email.
- The Sponsor will provide in the email the protocol number and trial name, name of the requester, reason for unblinding, participant's PIN, participant's randomization code and timeline to receive the unblinded information.
- The Sponsor will provide the unblinded information on the e-SUSAR website form.

This information will not be forwarded to the trial team and kept in the JRO site file.

Visit 1: Screening Assessment

The investigator will contact the participants to follow a standard diet for the 24 hours prior to the scheduled visit day and to avoid alcohol and strenuous exercise. They will fast from 20:00 on the night before the study visit and drink only water. The following screening procedures will be carried out:

- Physical examination.
- Vital signs (heart rate (HR) and blood pressure (BP).
- Medical history and co-morbidities.

- Concomitant medications.
- Urine pregnancy test for women of childbearing potential.
- Weight and height.
- Blood tests:
    - ✓ Haematology: full blood count, urea and electrolytes.
    - ✓ Serum biochemistry: renal, liver and thyroid function, glucose, lipids, HbA1c.
    - ✓ Gut hormones and adipokines.
- Meal test will be assessed using our established protocols [25]. Fasted baseline bloods and subjective appetite (assessed using validated visual analogue scores) will be undertaken. At time 0 (t0) a 500 kcal liquid meal will be consumed within 10 minutes. Repeated blood samples and appetite assessments will be made at 15 and 30 minutes. Suboptimal active GLP-1 response is defined as a ≤ 2-fold increase in active GLP-1 circulating levels between time 0 and time 30 minutes.

Once all data related to the screening visit has been obtained, the investigator will review the data to ensure that the participants is eligible to take part in the BARI-OPTIMISE trial. Subjects who are screen-failure will not be re-screened and enrolled in the trial. All screening procedures will be carried out as specified in the schedule of assessments (Appendix 1). An investigator will call the patient to notify of the outcome of screening, answer their questions and explain possible next steps (depending on outcome).

Visit 2: Baseline Assessment (Day 1)

All participants who have consented for the trial must meet all the inclusion and exclusion criteria as set out in section 6.1 and 6.2. No eligibility waivers or deviations will be permitted. Prior to the scheduled assessment, the investigator will contact the participants to follow a standard diet for the 24 hours prior to the visit day and to avoid alcohol and strenuous exercise. They will fast from 20:00 on the night before the study visit and drink only water. The investigator will also remind participants to complete and bring to the visit the food diary that would have been sent to them (electronically and/or via post) in advance and in preparation for the baseline data collection. The following baseline assessments will be carried out:

- Sociodemographic data.
- Physical examination.
- Vital signs (HR and BP).
- Urine pregnancy test for women of childbearing potential.
- Weight.
- BIA.
- Dual-energy x-ray absorptiometry (DXA) scanning.
- Blood tests:
    - ✓ Haematology: full blood count.
    - ✓ Serum biochemistry: renal, liver and thyroid function, glucose, lipids, HbA1c.
    - ✓ Gut hormone and adipokines.
- Physical function assessments:
    - ✓ 6 minute walk-test (6MWT)
    - ✓ sit-to-stand (STS) test
    - ✓ Hand-grip test

- Physical activity levels using the International Physical Activity Questionnaire (IPAQ).
- HRQoL questionnaires:
  - ✓ Impact of weight on quality of life-lite (IWQOL-Lite)
  - ✓ Beck depression inventory (BDI).
- Health Economics questionnaires:
  - ✓ Adapted Client Service Receipt Inventory (CSRI) [26]
  - ✓ EuroQol-5D (EQ-5D) [27, 28]
- Meal test: fasted baseline bloods and subjective appetite (assessed using validated visual analogue scores) will be undertaken. At t0 a 500 kcal liquid meal will be consumed within 10 minutes. Repeated blood sampling and appetite assessments will be undertaken (T15, T30, T60, T90, T120, T150 and T180). Glucose, gut hormones and adipokines will be monitored using our established protocols [25].
- Counselling on lifestyle modification (500-kcal deficient diet and 150 minute of physical activity/week).
- Collection of food diary (3 days of food intake: 2 weekdays, 1 weekend day) completed at home by participants.
- Distribution of:
  - ✓ New food diary (3 days of food intake: 2 weekdays, 1 weekend day)
  - ✓ Drug diary
- Subcutaneous injection training. Participants will be contacted by the investigator to assess their injection technique. They will be offered additional injection training by the research investigator as required.

Treatment procedures

24-week trial of once daily subcutaneously injected escalating liraglutide 3.0 mg or placebo as an adjunct to diet and exercise. Participants will receive on-going counselling for diet and exercise throughout the trial period by healthcare professionals. Liraglutide is a licensed drug. Novo Nordisk will supply liraglutide 3.0 mg and placebo as a solution for injection in a 3 ml pre-filled dial-a-dose pen-injector containing placebo or liraglutide 3.0 mg (6 mg/ml). Study medication will only be administered once all visit 2 assessments have been completed and the relevant safety reports have been obtained. All eligibility criteria have to be fulfilled and no exclusion criteria must be identified.

Subsequent assessments

Visits 3-6: Intervention Phase (Days 15, 29, 57 & 113) (+/- 3 days)

The following will be assessed at each of the trial visits:

Targeted physical examination.

- Vital signs (HR and BP).
- Weight.
- BIA.
- Concomitant medications.
- Drug diary review.
- Adverse event (AE) review.
- Review of glucose monitoring at home.
- Urine pregnancy test for women of childbearing potential.
- Collection of food diary and distribution of new one.

- Counselling on lifestyle modification.
- Review of:
    - ✓ Drug diary
- Injection training will be repeated at Visit 3 (day-15), as needed.

Visit 7: End-of-Treatment Assessment (Day 169 following the last dose) (+/- 3 days)

Prior to the scheduled assessment, the investigator will contact the participants to follow a standard diet for the 24 hours prior to the visit day and to avoid alcohol and strenuous exercise. They will fast from 20:00 on the night before the study visit and drink only water. The following assessments will be carried out:

- Targeted physical examination.
- Vital signs (HR and BP).
- Weight.
- BIA.
- DXA scanning.
- Concomitant medications.
- AE review.
- Urine pregnancy test for women of childbearing potential.
- Blood tests:
    - ✓ Haematology: full blood count.
    - ✓ Serum biochemistry: renal, liver and thyroid function, glucose, lipids, HbA1c.
    - ✓ Gut hormone and adipokines.
- Physical function assessments:
    - ✓ 6 minute walk-test (6MWT)
    - ✓ sit-to-stand (STS) test
    - ✓ Hand-grip test
- Physical activity levels using the International Physical Activity Questionnaire (IPAQ).
- HRQoL questionnaires:
    - ✓ Impact of weight on quality of life-lite (IWQOL-Lite)
    - ✓ Beck depression inventory (BDI).
- Health Economics questionnaires:
    - ✓ Adapted Client Service Receipt Inventory (CSRI)
    - ✓ EuroQol-5D (EQ-5D)

- Collection of:
    - ✓ Food diary
    - ✓ Drug diary
- Meal test: fasted baseline bloods and subjective appetite (assessed using validated visual analogue scores) will be undertaken. At t0 a 500 kcal liquid meal will be consumed within 10 minutes. Repeated blood sampling and appetite assessments will be undertaken (T15, T30, T60, T90, T120, T150 and T180) Glucose, gut hormones and adipokines will be monitored using our established protocols [25].

End of Trial Assessment (Day 197) (+/- 3 days)

The investigator will call the participant 4 weeks after the last treatment injection to review and collect any adverse event that might have occurred since end of treatment. This final assessment will be carried out over the phone.

Participants who withdraw before the end of the trial will be asked to attend for their end-of-trial visit (as soon as possible after withdrawal) and their data will be imputed using last observation carry forward (LOCF) method. Withdrawn participants will not be replaced and no follow-up assessment will be carried out.

Name and description of all drugs used in the trial

Treatment of subjects

The investigational medicinal product in this trial is liraglutide 3.0 mg. Liraglutide is a licensed drug. A placebo that is visually identical to liraglutide 3.0 mg will be used as a comparator. Novo Nordisk will supply Liraglutide 3.0 mg and placebo.

9.2 Concomitant medications

Concomitant medications will be recorded in the participant's medical records/CRF/eCRF and reviewed at each trial visit. For participants with T2D taking anti-diabetic medications, such as sulfonylureas, medication review will be throughout the trial period. Glucose monitoring will be commenced one week prior to randomisation and continued throughout the study period and the anti-diabetic medication will be adjusted accordingly. Careful monitoring of symptoms of hypoglycaemia will be carried out throughout the trial. The use of insulin, DPPIV-inhibitors, GLP-agonist or any medication known to cause weight gain or weight loss will not be permitted in this study. Also, people who are on these medications will not be recruited into the trial.

Liraglutide causes a delay in gastric emptying, and thereby has the potential to impact the absorption of concomitantly administered oral medications. Further details of interaction with other medicinal products and other forms of interaction with the IMP can be found in the SmPC.

Investigational Medicinal Product

Name and description of Investigational Medicinal Product

Name:	Liraglutide 3.0 mg
Composition:	Liraglutide, Disodium phosphate dehydrate, Propylene glycol, Phenol, Water for injection, Sodium hydroxide, Hydrochloric acid.
Manufacturer:	Novo Nordisk

Name and description of Non-Investigational Medicinal Product

Name:	Placebo
Composition	Disodium phosphate dehydrate, Propylene glycol, Phenol, Water for injection, Sodium hydroxide, Hydrochloric acid.
Manufacturer:	Novo Nordisk

Dosages, modifications and method of administration

Dose escalation schedule of liraglutide 3.0 mg/ placebo is as following:

	Dose (pen-setting once daily subcutaneously)	Weeks
**Dose escalation 4 weeks**	0.6 mg	1
	1.2 mg	2
	1.8 mg	3
	2.4 mg	4
**Maintenance dose**	3.0 mg	5 – 24

At the end of baseline visit, after randomisation, participants will be trained and instructed to inject liraglutide 3.0 mg /placebo once daily at any time of day, without regard to the timing of meals, but ideally establish a routine for the same time of each day. Liraglutide 3.0 mg/placebo can be injected subcutaneously in the abdomen, thigh, or upper arm. The injection site and timing can be changed without dose adjustment, but it is important to try and stick to similar timing each day. If a dose is missed, participants will be instructed to resume the once-daily regimen as prescribed with the next scheduled dose. An extra dose or increase in dose should not be taken to make up for the missed dose. If more than 3 days have elapsed since the last dose of liraglutide 3.0 mg or placebo patients will be instructed to reinitiate liraglutide 3.0 mg or placebo at 0.6 mg daily pen setting and follow the dose escalation schedule till the scheduled end of the treatment period (i.e. the 24-week treatment period will not restart). Participants will be invited to attend pharmacy to collect liraglutide 3.0 mg/placebo pre-filled pens and the dispensing schedule will be as follow:

- Baseline visit: 5 pens (i.e. 1 box)
- Week 4 follow-up visit: 5 pens (i.e. 1 box)
- Week 8 follow-up visit: 13 pens (i.e. 2 boxes plus 3 pens)
- Week 17 follow-up visit: 5 pens (i.e. 1 box)

Drug accountability (liraglutide 3.0 mg/ placebo)

The drug aspects of the trial will be delegated by the CI to a delegated Lead Pharmacist. Novo Nordisk will supply liraglutide 3.0 mg and placebo. UCLH pharmacy will be responsible for storage and dispensing of liraglutide 3.0 mg and placebo according to handling and storage instructions as reported in the Summary Of Drug Arrangements (SODA).

A log of all received, used, partly used and unused trial products will be kept. The trial products will be dispensed to each participant as required according to treatment group. Proper storage conditions (outlined below) will be available and the temperature will be evaluated and recorded at least every working day. No trial product should be dispensed to any person not enrolled in the trial.

<u>Not in use</u>: The liraglutide 3.0 mg/placebo will be stored in a refrigerator at a temperature between +2°C and +8°C, protected from light and kept away from the freezer compartment. Under these conditions, the shelf life of liraglutide 3.0 mg/placebo pre-filled pens is of 30 months.

<u>In use</u>: After first opening the liraglutide 3.0 mg/placebo pre-filled pen can be stored for one month at temperatures below +30°C or in a refrigerator between +2°C and +8°C. The drug should never be kept at temperatures higher than 30°C; the cap should be kept on the pen to protect from light at all times.

All used/unused IMPs will be returned to site pharmacy, to be then updated in the drug accountability log in the pharmacy site file. Drug destruction will be conducted, once authorised by the sponsor and in accordance with local practice, or returned to Novo Nordisk and this will be documented in the drug destruction log in the hospital pharmacy file.

Detailed instructions are contained in the summary of drug arrangements.

Dose modifications

If participants are unable to tolerate an increased dose during dose escalation, delaying dose escalation for approximately one additional week will be considered. Liraglutide 3.0 mg/placebo will be discontinued, however, if a participant cannot tolerate the 3.0 mg dose, as efficacy has not been established at lower doses. Any dosage changes will be logged in a drug diary by participants and this will be reviewed at each trial visits.

Assessment of compliance

Compliance includes both adherences to IMP and Protocol study procedures. Subjects will be provided with a drug diary to record their home-dosing. Any missed dosage will be logged in the drug diary. This is a way to assess subject's compliance to the treatment. This will be reviewed at each trial visit. Percentage of IMP compliance acceptable for patient to continue on the trial is 80%. Non-compliance will be documented by the investigator in the medical notes and reported to the sponsor. Persistent non-compliance may lead to subject withdrawal from the study.

Recording and reporting of adverse events and reactions

Collection, recording and reporting of adverse events (including serious and non-serious events and reactions) to the sponsor will be completed according to the sponsor's SOP (INV/S05).

Definitions

**Term**	**Definition**
Adverse Event (AE)	Any untoward medical occurrence in a patient or clinical trial participant administered a medicinal product and which does not necessarily have a causal relationship with this treatment. *Therefore an AE can be any unfavourable or unintended change in the structure (signs), function (symptoms) or chemistry (laboratory data) in a participant to whom an IMP or procedural intervention has been administered, including occurrences which are not necessarily caused by or related to that product.*
Adverse Reaction (AR)	Any untoward and unintended response in a participant to an investigational medicinal product which **is related** to any dose administered to that participant.<br><br>*This includes medication errors, uses outside of protocol (including misuse and abuse of product)*
Serious Adverse Event (SAE), Serious Adverse Reaction (SAR) or Unexpected Serious	Any adverse event, adverse reaction or unexpected adverse reaction, respectively, that:<br><br>• results in death,

Adverse Reaction	• is life-threatening*,<br>• requires hospitalisation or prolongation of existing hospitalisation**,<br>• results in persistent or significant disability or incapacity, or<br>• consists of a congenital anomaly or birth defect<br>• Raises suspicion of transmission of infectious agents
*A life- threatening event refers to an event in which the participant was at risk of death at the time of the event; it does not refer to an event which hypothetically might have caused death if it were more severe.<br>** Hospitalisation is defined as an in-patient admission, regardless of length of stay. Hospitalisation for pre-existing conditions, including elective procedures do not constitute an SAE.	
Unexpected adverse reaction	An adverse reaction the nature and severity of which is not consistent with the information about the medicinal product in question set out:<br>(a) in the case of a product with a marketing authorization, in the summary of product characteristics for that product,<br>(b) in the case of any other investigational medicinal product, in the investigator's brochure relating to the trial in question.
Suspected Unexpected Serious Adverse Reaction (SUSAR)	An unexpected adverse reaction which is also categorised as serious.
Important Medical Event	These events may jeopardise the participant or may require an intervention to prevent one of the above characteristics/consequences. Such events should also be considered 'serious'.

Recording adverse events

Adverse events will be recorded by the investigator at each visit post-baseline. AEs will be documented in the medical notes in the first instance, and CRF following consent. When recording an adverse event, clinical symptoms and a simple, brief description of the event, including dates as appropriate, should be reported. Clinically significant abnormalities in the results of objective tests (e.g. laboratory variables) will also be recorded as AEs in the medical notes and if are assessed as serious they will also be recorded on the AE log and in the eCRF. If these events are not expected as part of disease or IMP, these will be recorded as unexpected. All adverse events will be recorded until 4 weeks after the end of treatment. At the end-of-trial assessment (i.e. four weeks after participant's end-of-treatment visit) a trial investigator will call the participants to collect any adverse event up until that point and this will be recorded in the medical notes and CRF following the same procedure as outlined above.

Assessments of Adverse Events

Each adverse event will be assessed for the following criteria:

Severity

Category	Definition
Mild	The AE does not interfere with the participant's daily routine, and does not require further intervention; it causes slight discomfort.
Moderate	The AE interferes with some aspects of the participant's routine, or requires further intervention, but is not damaging to health; it causes moderate discomfort.
Severe	The AE results in alteration, discomfort or disability which is clearly damaging to health.

Causality

The assessment of relationship of adverse events to the administration of liraglutide 3.0 mg/placebo is a clinical decision based on all available information at the time of the completion of the eCRF. The following categories will be used to define the causality of the adverse event:

Category	Definition
Definitely	There is clear evidence to suggest a causal relationship, and other possible contributing factors can be ruled out.
Probably	There is evidence to suggest a causal relationship, and the influence of other factors is unlikely.
Possibly	There is some evidence to suggest a causal relationship (e.g. the event occurred within a reasonable time after administration of the trial intervention). However, the influence of other factors may have contributed to the event (e.g. the participant's clinical condition, other concomitant events).
Unlikely	There is little evidence to suggest there is a causal relationship (e.g. the event did not occur within a reasonable time after administration of the trial intervention). There is another reasonable explanation for the event (e.g. the participant's clinical condition, other concomitant treatments).
Not related	There is no evidence of any causal relationship.
Not Assessable	Unable to assess on information available.

11.3.3 Expectedness

Category	Definition
Expected	An adverse event that is classed in nature as serious and which is consistent with the information about liraglutide 3.0 mg listed in the SmPC.
Unexpected	An adverse event that is classed in nature as serious and which is not consistent with the information about liraglutide 3.0 mg listed in the SmPC.

The reference document to be used to assess expectedness against the IMP is the SmPC.

Seriousness

Seriousness as defined for an SAE in section 0.0.0. Collection, recording and reporting of adverse events (including serious and non-serious events and reactions) to the sponsor will be completed according to the sponsor's SOP (INV/S05).

Pregnancy

If a female participant or the female partner of a male participant becomes pregnant at any point during the trial, a completed trial specific Pregnancy Reporting Form will be preferably emailed to the Sponsor SAE@ucl.ac.uk and/or faxed on **020 3108 2312,** within 24 hours of his / her becoming aware of the event in line with the Sponsors SOP (JRO/INV/S05). The Chief Investigator will respond to any queries raised by the sponsor as soon as possible.

The Sponsor must be kept informed of any new developments involving the pregnancy through the completion of a follow-up Pregnancy Reporting Form. Any pregnancy that occurs in a female trial subject during a clinical trial should be followed to termination or to term.
Consent to report information regarding the pregnancy [include follow-up of a child born if applicable] must be obtained from the pregnant participant [include partner if applicable]. A trial-specific pregnancy monitoring information sheet and informed consent form for trial participants [include the partners of trial participants if applicable] must be used for this purpose.

With consent, additional information regarding the pregnancy will be collected and reported to the Sponsor, the Sponsor will advise on the length of follow up of the pregnancy/ child on a case by case basis.

Overdose

In the event of an accidental or intentional overdose by a trial participant, the investigators will immediately inform the CI and the Sponsor's office. Overdose can be observed from the drug diary or reported by participants. The deviation log will be completed and the medical notes, eCRF, AE log will be updated to reflect this information. In the event that the overdose is associated with an S/AE, the two events will be linked. In the event of an AE associated with an overdose, a SAE report form will be completed detailing the AE and the overdose details. The investigators will justify whether patients should remain or withdrawn from the trial. Resultant symptoms will be treated as per routine clinical care.

Data management and quality assurance

Confidentiality

The CI will act as the custodian for the trial data. All data will be handled in accordance with the UK Data Protection Act 1998. Each participant will be given a unique trial identification number at the start and used on their records instead of their name. The master list linking participants' name and the trial identification number will be kept in a password-protected computer. This way, participants' personal identity and data collected in the study cannot be connected by anyone outside the study team. The eCRFs will not bear the participant's name or other personal identifiable data. The subject's initials, date of birth and trial identification number will be used for identification and this will be clearly explained to participants in the PIS. Identifying participant information will be kept

separate from research data. Consent forms and other paper records will be stored in locked filing cabinets in swipe-card accessed offices.

Data collection tools and source document identification

Data will be collected using CRF. Source data will be accurately transcribed on to the CRF. Examples of source documents are medical records that included laboratory and other clinical reports. A source document list will be implemented prior to the start of the trial to identify data to be recorded firstly into source documents, such as medical notes and then transcribed onto the CRF.

The database and CRF will be designed in conjunction so that data captured are complete, accurate, reliable and consistent. The delegation log will identify all those personnel with responsibility for data handling including those who have access to the trial database.
The Investigators are responsible for ensuring the accuracy of all the data entered in the eCRFs. Source data and CRF data will be checked as being accurate, complete, reliable and consistent before it is entered onto the database by individuals delegated the responsibility outlined in the delegation log. This will include

- Screening data verification prior to randomisation to ensure patients fulfil the inclusion/exclusion criteria.
- Ensuring that all AEs are reported and recorded.
- Queries relating to eCRF entries are corrected within a suitable time frame.
- CRF will be checked for errors before being deemed as complete and this process will be documented.

A Data Manager will be appointed to ensure that appropriate corrections, additions, or deletions are made, dated, explained and initialled by the Investigator or by a member of the Investigator's trial staff who is authorised to initial CRF changes for the Investigator. A Data and Safety Monitoring Plan (DSMP) will outline trial procedures to be undertaken in ensuring data and safety monitoring throughout the lifespan of the Trial.

Completing Case Report Forms

All CRFs will be completed and signed by staff that are listed on the staff delegation log and authorised by the CI to perform this duty. The CI will be responsible for the accuracy of all data reported in the CRF.

Data handling and analysis

All data will be collected from participants in accordance with the participant consent form and PIS. The data will be appropriately sent to an appointed Data Manager and Trial Statistician for processing and statistical analysis and the Sponsor will act as the data controller of such data for the study. Data will be processed, stored and disposed of in accordance with all applicable legal and regulatory requirements including the Data Protection Act 1998 and any amendments thereto. eCRF and questionnaires will be stored in locked filing cabinets controlled by the CI. The database as well as laptop and/or PC will be password protected. Information regarding database backup and storage will be documented. The data will not be transferred to any party not identified in this protocol and will not be processed and/or transferred other than in accordance with the participant's consent.

Statistical Considerations

is the trial statistician who will be responsible for all statistical aspects of the trial from design through to analysis and dissemination.

Outcomes

Primary Outcome

The primary outcome of this trial is %WL from the baseline visit to the end of treatment visit at 24 weeks. Percentage weight loss will be calculated using the following formula: %WL = [(weight at the baseline visit–weight at the end of the 24-week treatment period)/ weight at the baseline visit] x 100, measured at the end of treatment.

Secondary Outcomes

The secondary outcomes of this trial are:

1. To compare changes in fat (%), lean body mass (%) and bone density from baseline visit to end of 24-week treatment period, assessed using DXA scanning, between liraglutide 3.0 mg and placebo.
2. To compare changes in circulating fasted glucose, insulin, HbA1c and leptin, and meal-stimulated glycaemic index, gut hormones and appetite response from baseline visit to end of 24-week treatment period between liraglutide 3.0 mg and placebo.
3. To compare changes in HRQoL (aggregate scores) assessed using IWQOL-Lite and BDI from baseline visit to end of 24-week treatment period between liraglutide 3.0 mg and placebo.
4. To compare changes in characteristics of attitude and symptom of depression (aggregate scores) assessed using BDI from baseline visit to end of 24-week treatment period between liraglutide 3.0 mg and placebo.
5. To compare changes in physical fitness assessed using 6MWT (distance covered in m^2), STS test (s) and handgrip test (kg) from baseline visit to end of 24-week treatment period between liraglutide 3.0 mg and placebo.
6. To compare changes in physical activity level assessed using IPAQ from baseline visit to end of 24-week treatment period between liraglutide 3.0 mg and placebo.
7. To compare changes in healthcare service usage from baseline visit to end of 24-week treatment period between liraglutide 3.0 mg and placebo.

Sample size calculation

Using the 20-week data from the SCALE trial [23], 66 patients (33 per group) will be needed to detect a difference of 5%WL using a two-sample t-test, assuming a common SD of 5.4%, 90% power, 5% statistical significance allowing for a 20% drop-out rate. We anticipate screening between 100 and 150 patients with less than 20% weight loss at one year or more post-surgery. We will consent, recruit and randomise the first 70 patients who fulfil the inclusion and exclusion criteria.

Planned recruitment rate

Participants will be recruited from UCLH, Homerton and Whittington Bariatric Centres. These units have been established for >5 years and undertake >800 primary RYGB and/or SG per year and follow up their patients indefinitely. At least 20% of patients undergoing RYGB and SG experience poor weight loss providing approximately 160 potentially eligible patients. We anticipate recruiting at least 20 participants per month and to complete recruitment within a 6-month period.

Statistical analysis plan

A detailed analysis plan will be drawn up prior to database lock or seeing any data.

Summary of baseline data and flow of patients

A consort diagram will be presented. Patient characteristics will be described using means (SDs) or medians (interquartile range) for continuous measures and proportions for categorical measures. These values will be presented by randomisation group.

Primary outcome analysis

The mean difference in %WL at 24 weeks between the groups will be analysed using linear regression, adjusting for stratification variables and any baseline variables which are not balanced between the groups. Mean difference in %WL will be reported with 95% confidence interval. The assumptions of the model will be checked, and a suitable transformation/non-parametric method will be used where the assumptions are not met.

All available data will be analysed as randomised. Bias due to missing data will be investigated and dealt as appropriate.

Secondary outcome analysis

The results of the secondary analysis will be treated as exploratory.

Continuous outcomes will be analysed using separate linear regression models, adjusting for stratification variables and any baseline variables which are not balanced between the groups. Mean differences in each outcome will be reported with 95% confidence intervals. The assumptions of each model will be checked, and a suitable transformation/non-parametric method will be used where the assumptions are not met.

To evaluate the economic impact of the trial intervention, we will calculate the cost-effectiveness from an NHS and personal social services (PSS) perspective, relative to usual care. The analysis will be based on per-participant intervention costs, and NHS/PSS resource use and HRQoL assessed retrospectively in the trial between baseline and end of the 24-week treatment period. We will calculate the incremental cost per quality-adjusted life year (QALY) gained for the within-trial period. We will run deterministic and probabilistic sensitivity analyses.

Interim Analysis

None planned.

Other statistical considerations

Any deviations from the original statistical plan will be described and justified in the protocol and/or in the final report, as appropriate.

Ethics and regulatory requirements

The trial will be conducted in compliance with the principles of the Declaration of Helsinki (1996), the principles of International Conference on Harmonisation Good Clinical Practice (ICH GCP) and in accordance with all applicable regulatory requirements including but not limited to the Research Governance Framework and the Medicines for Human Use (Clinical Trial) Regulations 2004, as amended in 2006 and any subsequent amendments. NHS management permission will be obtained from the UCLH JRO who will also undertake data monitoring and provide Sponsorship for the trial.

Ethical approval for this study will be obtained from the Health Research Authority (via the Integrated Research Application System) including review by an NHS Research Ethics Committee (REC) and from the Medicines and Healthcare products Regulatory Agency (MHRA) for Clinical Trial Authorisation. The CI will submit a final report at conclusion of the trial to the REC and the MHRA.

The Sponsor will ensure that trial protocol, participant information sheet, consent form, GP letter and submitted supporting documents have been approved by the appropriate REC, prior to any participant recruitment. The protocol, all supporting documents and agreed documents, will be documented and submitted for ethical and regulatory approval as required. Amendments will not be implemented prior to receipt of the required approval (s).

Before any NHS site may be opened to recruit participants, the (CI) Investigator/Principal Investigator (PI) or designee must receive a Trust confirmation of capacity and capability. It is the responsibility of the CI/ PI or designee at each site to ensure that all subsequent amendments gain the necessary approvals, including NHS Permission (where required) at the site and the HRA approvals. This does not affect the individual clinician's responsibility to take immediate action if thought necessary to protect the health and interest of individual participants.

Within 90 days after the end of the trial, the CI/Sponsor will ensure that the main REC is notified that the trial has finished. If the trial is terminated prematurely, those reports will be made within 15 days after the end of the trial. The CI will supply the Sponsor with a summary report of the trial, which will then be submitted to the REC within 1 year after the end of the trial.

Statement of compliance

This trial will be conducted in compliance with the protocol, the UK Regulations, EU GCP and applicable regulatory requirement (s).

References

1. Angrisani, L., et al., Bariatric Surgery Worldwide 2013. Obes Surg, 2015. 25(10): p. 1822-32.
2. NICE, NICE Clinical Guidelines [CG189]: Obesity: identification, assessment and management. https://www.nice.org.uk/guidance/cg189, 2014.
3. Manning, S., et al., Early postoperative weight loss predicts maximal weight loss after sleeve gastrectomy and Roux-en-Y gastric bypass. Surg Endosc, 2015. 29(6): p. 1484-91.
4. de Hollanda, A., et al., Patterns of Weight Loss Response Following Gastric Bypass and Sleeve Gastrectomy. Obes Surg, 2015. 25(7): p. 1177-83.
5. Batterham, R.L. and D.E. Cummings, Mechanisms of Diabetes Improvement Following Bariatric/Metabolic Surgery. Diabetes Care, 2016. 39(6): p. 893-901.
6. Jimenez, A., et al., Long-term effects of sleeve gastrectomy and Roux-en-Y gastric bypass surgery on type 2 diabetes mellitus in morbidly obese subjects. Ann Surg, 2012. 256(6): p. 1023-9.
7. Arterburn, D.E., et al., A multisite study of long-term remission and relapse of type 2 diabetes mellitus following gastric bypass. Obes Surg, 2013. 23(1): p. 93-102.
8. Lee, M.H., et al., Predictors of long-term diabetes remission after metabolic surgery. J Gastrointest Surg, 2015. 19(6): p. 1015-21.
9. Coleman, K.J., et al., Long-term Microvascular Disease Outcomes in Patients With Type 2 Diabetes After Bariatric Surgery: Evidence for the Legacy Effect of Surgery. Diabetes Care, 2016. 39(8): p. 1400-7.
10. Laurino Neto, R.M., et al., Comorbidities remission after Roux-en-Y Gastric Bypass for morbid obesity is sustained in a long-term follow-up and correlates with weight regain. Obes Surg, 2012. 22(10): p. 1580-5.
11. Sundbom, M., et al., Substantial Decrease in Comorbidity 5 Years After Gastric Bypass: A Population-based Study From the Scandinavian Obesity Surgery Registry. Ann Surg, 2016.
12. Lassailly, G., et al., Bariatric Surgery Reduces Features of Nonalcoholic Steatohepatitis in Morbidly Obese Patients. Gastroenterology, 2015. 149(2): p. 379-88; quiz e15-6.
13. Caiazzo, R., et al., Roux-en-Y gastric bypass versus adjustable gastric banding to reduce nonalcoholic fatty liver disease: a 5-year controlled longitudinal study. Ann Surg, 2014. 260(5): p. 893-8; discussion 898-9.
14. Mohos, E., et al., Quality of life, weight loss and improvement of co-morbidities after primary and revisional laparoscopic roux Y gastric bypass procedure-comparative match pair study. Obes Surg, 2014. 24(12): p. 2048-54.
15. Raoof, M., et al., Health-Related Quality-of-Life (HRQoL) on an Average of 12 Years After Gastric Bypass Surgery. Obes Surg, 2015. 25(7): p. 1119-27.
16. Manning, S., A. Pucci, and R.L. Batterham, Roux-en-Y gastric bypass: effects on feeding behavior and underlying mechanisms. J Clin Invest, 2015. 125(3): p. 939-48.
17. Manning, S., A. Pucci, and R.L. Batterham, GLP-1: a mediator of the beneficial metabolic effects of bariatric surgery? Physiology (Bethesda), 2015. 30(1): p. 50-62.
18. Dirksen, C., et al., Gut hormones, early dumping and resting energy expenditure in patients with good and poor weight loss response after Roux-en-Y gastric bypass. Int J Obes (Lond), 2013. 37(11): p. 1452-9.
19. Gerner, T., et al., The post-prandial pattern of gut hormones is related to magnitude of weight-loss following gastric bypass surgery: a case-control study. Scand J Clin Lab Invest, 2014. 74(3): p. 213-8.
20. Blackman, A., et al., Effect of liraglutide 3.0 mg in individuals with obesity and moderate or severe obstructive sleep apnea: the SCALE Sleep Apnea randomized clinical trial. Int J Obes (Lond), 2016. 40(8): p. 1310-9.
21. Pajecki, D., et al., Short-term use of liraglutide in the management of patients with weight regain after bariatric surgery. Rev Col Bras Cir, 2013. 40(3): p. 191-5.
22. Perna, S., et al., Liraglutide and obesity in elderly: efficacy in fat loss and safety in order to prevent sarcopenia. A perspective case series study. Aging Clin Exp Res, 2016. 28(6): p. 1251-1257.
23. Pi-Sunyer, X., et al., A Randomized, Controlled Trial of 3.0 mg of Liraglutide in Weight Management. N Engl J Med, 2015. 373(1): p. 11-22.
24. Rondanelli, M., et al., Twenty-four-week effects of liraglutide on body composition, adherence to appetite, and lipid profile in overweight and obese patients with type 2 diabetes mellitus. Patient Prefer Adherence, 2016. 10: p. 407-13.

25. Youssef, A., et al., Differential effects of laparoscopic sleeve gastrectomy and laparoscopic gastric bypass on appetite, circulating acyl-ghrelin, peptide YY3-36 and active GLP-1 levels in non-diabetic humans. Obes Surg, 2014. 24(2): p. 241-52.
26. Beecham, J. and M. Knapp, Costing psychiatric interventions, in G. Thornicroft (ed.) Measuring Mental Health Needs. 2nf edition ed. 2001: Gaskell.
27. EuroQol, G., EuroQol--a new facility for the measurement of health-related quality of life. Health Policy, 1990. 16(3): p. 199-208.
28. Brooks, R., EuroQol: the current state of play. Health Policy, 1996. 37(1): p. 53-72.

BARI-OPTIMISE SUMMARY OF AMENDMENTS LOG

Amendment Number	Date Submitted	Where Submitted			Classification		Purpose of Amendment	Date Approved (Substantial Amendments Only)		
		REC	HRA	MHRA	Substantial	Non Substantial		REC	HRA	MHRA
1	20/04/2018	✓	✓	✓	✓		To update protocol on changes requested by competent Authorities during initial review, to notify of change in PI at PIC and to submit a patient's booklet	13/06/18	14/06/18	12/06/18
2	07/02/2019	✓	✓	N/A	✓		Inclusion of new recruiting site	20/03/19	20/03/19	N/A
NS#1	13/09/2019	N/A	✓	N/A		✓	Extension of Trial end date	N/A	18/09/2019	N/A

Statistical Analysis Plan

BARI-OPTIMISE Trial

Statistical Analysis Plan

VERSION 4

5 Feb 2021

Contents

1 Study Summary 3
2 List of Abbreviations 5
3 Introduction 6
3.1 Purpose of the statistical analysis plan 6
3.2 Authorship 6
3.3 Organization of Data and Analyses 6
4 Summary of Quantitative Trial Data 7
4.1 Observation times 7
4.2 Summary of Outcome Measures 7
4.2.1 Primary outcome 7
4.2.2 Secondary outcome 8
5 Analyses 8
5.1 Recruitment and Retention 8
5.2 Description of t Baseline Characteristics 10
5.3 Analysis of the Primary Outcome 10
5.3.1 Model checking 11
5.4 Secondary analyses 112
5.5 Missing data 123
5.6 Reporting 13
6 Reference 14
7 Signatures 14

866 1. 1 Study Summary

Title

A double-blinded, randomised, placebo-controlled trial of liraglutide 3.0 mg in patients with poor weight-loss and a suboptimal glucagon-like peptide-1 response following bariatric surgery.

Aims

To test the hypothesis that for patients with suboptimal nutrient-stimulated glucagon-like peptide-1 (GLP-1) response and poor weight loss following gastric bypass or sleeve gastrectomy, compared with placebo, 24-weeks of subcutaneous liraglutide 3.0 mg will result in:

- greater percentage weight loss (%WL)
- reduction in adiposity
- improvement in metabolic indices, physical function and health-related quality of life (HRQoL)

Outcome measures:

Primary outcome:

The primary objective of this trial is to compare the efficacy of 24-weeks of subcutaneous liraglutide 3.0 mg versus placebo administration, as an adjunct to diet and exercise, on %WL in participants with poor weight-loss and a sub-optimal active GLP-1 response following primary RYGB or SG at the end of the 24-week treatment period.

Secondary outcomes:

To compare the effect of 24-weeks of subcutaneous liraglutide 3.0 mg versus placebo administration as an adjunct to diet and exercise in participants with poor weight-loss and a sub-optimal active GLP-1 response following primary RYGB or SG, at the end of the 24-week treatment period, upon:

- Change in %WL over time (Week 2, 4, 8, 17 and 24)
- Change in fat, lean body mass and bone density.
- Change in circulating fasted glucose, insulin, HbA1c and leptin, and meal-stimulated glycaemic, gut hormone and appetite response.
- Change in HRQoL measures.
- Change in physical functional assessments.
- Safety as measured by the counts of adverse events.

Population:

Subjects with poor weight loss response (<20% of their total weight) following 1 year or more primary gastric bypass or primary sleeve gastrectomy.

Eligibility:

The inclusion and exclusion criteria can be found in the study

protocol.

Chief Investigator: Professor Rachael L. Batterham
Centre for Obesity Research
Division of Medicine
Rayne Building
5 University Street
London WC1E 6JF
Email: r.batterham@ucl.ac.uk

Sponsor representative: [redacted]

UCL Joint Research Office

1st floor, Maple House, 149 Tottenham Court Road,

London W1T 7NF

Postal address: Joint Research Office, UCL, Gower Street, London WC1E 6BT

2 List of Abbreviations

2.

BDI	Beck Depression Inventory
BIA	Bioelectrical Impedance Analyser
CONSORT	Consolidated Standards of Reporting Trials
CSRI	Client Service Receipt Inventory
DXA	Dual-energy x-ray absorptiometry
EQ-5D	EuroQol-5D
HbA1c	Glycosylated haemoglobin
HRQoL	Health Related Quality of Life
IPAQ	International Physical Activity Questionnaire
IWQOL-Lite	Impact of weight on quality of life-lite
6MWT	6-Minute Walk Test
NHS	National Health Service
PSS	Personal social services
RYGB	Roux-en-Y gastric bypass
SAP	Statistical analysis plan
SG	Sleeve gastrectomy
STS	Sit-to-stand test
T2D	Type 2 diabetes
WL	Weight loss

3. 3 Introduction

The BARI-OPTIMISE trial is a Phase IV, double-blinded, randomised, placebo-controlled trial. The aim of this trial is to determine whether 24-weeks of subcutaneous liraglutide 3.0 mg causes greater percentage weight loss (%WL), reduction in adiposity, improvement in metabolic indices, physical function and health-related quality of life (HRQoL) than placebo in patients with suboptimal nutrient-stimulated glucagon-like peptide-1 (GLP-1) response and poor weight loss following gastric bypass or sleeve gastrectomy.

Minor revisions were made to SAP V3 to include analyses of missing outcome data using self-reported weights and analyses of % weight loss as a categorical variable as part of the supportive analyses, before database lock.

1. 3.1 Purpose of the statistical analysis plan

This document contains details of the main quantitative, statistical, analyses for the BARI-OPTIMISE trial. These analyses will be pre-specified in order that they are not influenced by the collected trial data after unmasking. The statistical analysis plan (SAP) does not preclude the undertaking of further, ad-hoc, analyses, although the results of any such further analyses should be interpreted carefully. Furthermore, the SAP does not prevent the adaptation of any part of the trial analysis, should situations arise in which such adaptation is deemed necessary. The rationale for any such adaptation will be fully justified and transparent.

The SAP contains details of quantitative analyses only and does not describe any qualitative analyses.

2. 3.2 Authorship

3. 3.3 Organization of Data and Analyses

Following participant consent, screening and baseline measure collection, the randomisation procedure will be remotely carried out by the Sealed Envelope, an independent specialised company that provides 24/7 cover to undertake the randomisation and unblinding if required. The type of randomisation to be used is a stratified block randomisation with random block sizes. Subjects will be randomly assigned in a 1:1 ratio to receive either liraglutide 3.0 mg or placebo, stratified for type of surgical procedure and T2D status. This will be done by using either internet or telephone randomisation. Unmasking of randomisation group will occur once all data have been entered onto the trial database and checked, the database locked for analysis, the SAP has been finalized and approved and the primary analysis has been performed and the results replicated by another statistician. Stata

16 will be used for the analyses: the Stata programs and code to be used for statistical analyses will be prepared prior to the unmasking of data. Two statisticians will perform the analysis independently in relation to the primary outcome in order to ensure the accuracy of the results.

Prior to performing the main analyses, basic checks will be performed on the collected, masked, data to identify any anomalies. Each outcome (primary and secondary), key variables and baseline demographic variables will be checked for:

- missing values;
- values beyond an acceptable range;
- any other inconsistencies.

If missing values or other inconsistencies are present the corresponding data will be checked with the aid of the researchers and, where necessary, either corrected or deemed to be missing. Any such changes made to the dataset will be documented fully.

4. 4 Summary of Quantitative Trial Data

4. 4.1 Observation times

The times at which data are collected during the trial are as follows:

- Baseline (Week 0);
- Week 2;
- Week 4;
- Week 8;
- Week 17;
- Week 24.

5. 4.2 Summary of Outcome Measures

6. 4.2.1 Primary outcome

The primary outcome of this trial is %WL from the baseline visit to the end of treatment visit at 24 weeks. Percentage weight loss will be calculated using the following formula: %WL = [(weight at the baseline visit–weight at the end of the 24-week treatment period)/ weight at the baseline visit] x 100, measured at the end of trial.

4.2.1.1 Sample size

Using the 20-week data from the SCALE trial [1], 66 patients (33 per group) will be needed to detect a difference of 5%WL using a two-sample t-test, assuming a common SD of 5.4% for the intervention (liraglutide + lifestyle) and control groups (placebo + lifestyle), 90% power, 5% statistical significance allowing for a 20% drop-out rate and rounding up. Stata 15 (using sampsi command) was used to perform the sample size calculation [2].

7. 4.2.2 Secondary outcome

The secondary outcomes of this trial are to compare changes in:

1. Fat (%), lean body mass (%) and bone density from baseline visit to end of 24-week treatment period, assessed using DXA scanning and a bioelectrical impedance analyser (BIA), between liraglutide 3.0 mg and placebo.
2. Circulating fasted glucose, insulin, HbA1c and leptin, and meal-stimulated glycaemic, gut hormone and appetite response from baseline visit to end of 24-week treatment period between liraglutide 3.0 mg and placebo.
3. HRQoL (aggregate scores) assessed using CSRI, EQ-5D and IWQOL-Lite from baseline visit to end of 24-week treatment period between liraglutide 3.0 mg and placebo.
4. Characteristics of attitude and symptom of depression (aggregate scores) assessed using BDI from baseline visit to end of 24-week treatment period between liraglutide 3.0 mg and placebo.
5. Physical fitness assessed using 6MWT (distance covered in m), STS test(s) and handgrip test (kg) from baseline visit to end of 24-week treatment period between liraglutide 3.0 mg and placebo.
6. Physical activity level assessed using IPAQ from baseline visit to end of 24-week treatment period between liraglutide 3.0mg and placebo.
7. Healthcare service usage from baseline visit to end of 24-week treatment period between liraglutide 3.0mg and placebo.

5. 5 Analyses

8. 5.1 Recruitment and Retention

A CONSORT diagram [3] (Page 9 as an example) will be presented to provide a detailed description of patient numbers at each time point during the trial. In addition, a table summarizing the numbers of drop-outs at each stage of the trial and reasons for drop-out (if given) will be presented.

Consort diagram

CONSORT 2010 Flow Diagram

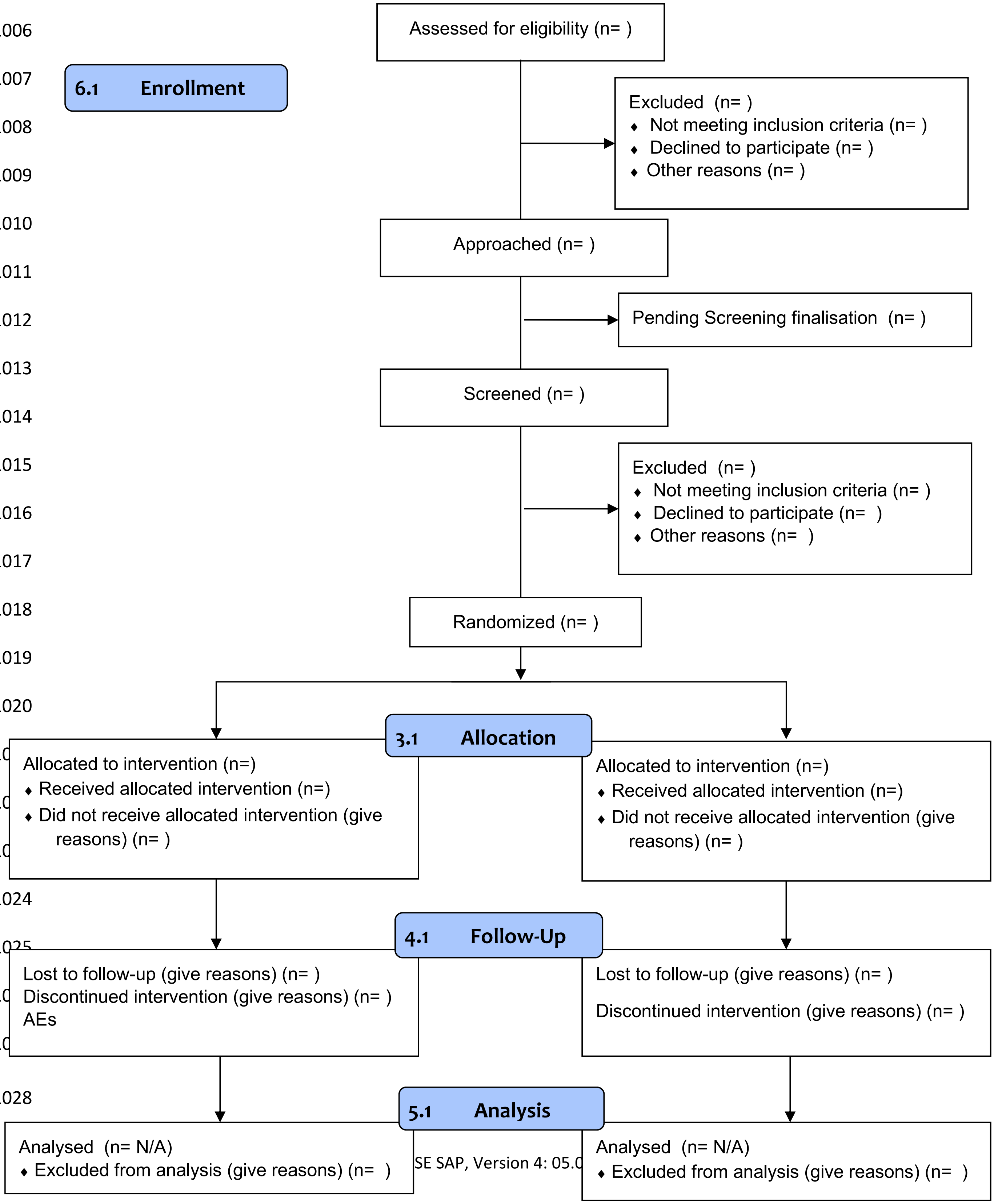

9. 5.2 Description of Baseline Characteristics

The demographic and other patient characteristics collected at baseline will be presented in a table, separated by trial arm. Categorical variables will be presented with the information (e.g. raw numbers and percentages) on each category. Reports of continuous variables will include mean (with standard deviation) and median (with interquartile range), as appropriate. Table 1 shows the layout of the table for baseline characteristics.

Table 1. Baseline characteristics

	**Liraglutide 3.0 mg**	**Placebo**
Age, years		
Gender (%Male)		
Ethnicity		

10. 5.3 Analysis of the Primary Outcome

The primary outcome is the mean difference in %WL at 24 weeks between the intervention and control groups. The main statistical analyses will estimate the difference in mean %WL between patients randomised to liragrutide + lifestyle and placebo + lifestyle.

Specifically, the following linear regression model will be fitted:

$$\%WL_i = \beta_0 + \beta_1 TRT_i + \beta_2 Baseline\_weight_i + \beta_3 Type_i + \beta_4 T2D_i + \epsilon_i$$

where:

- $\%WL_i$ is the weight loss at 24 weeks for the ith patient
- TRT_i is an indicator for the allocated treatment for the ith patient (0 = placebo, 1 = liraglutide)
- $Baseline\_weight_i$ is the weight at baseline for the ith patient
- $Type_i$ is an indicator for the type of surgical treatment the ith patient receives (0 = SG , 1 = RYGB) and is one of the stratification factors
- $T2D_i$ is an indicator for diabetes status for the ith patient (0 = no diabetes, 1 = have diabetes) and is one of the stratification factors
- β_0 is the regression intercept
- β_1 is the parameter of interest which quantifies the effect of treatment
- β_2 is the parameter of interest which quantifies the effect of baseline weight
- β_3 is the parameter of interest which quantifies the effect of type of surgical treatment
- β_4 is the parameter of interest which quantifies the effect of diabetes
- $\epsilon_i \sim N(0, \sigma_\epsilon^2)$ are the residuals

The results from this analysis will be presented in terms of a treatment difference (β_1), 95% confidence interval and a P-value. All analyses will be carried out comparing the intervention and control groups as randomised using all available data (intention to treat analysis). As part of a supportive analysis per protocol and as treated analysis will also be carried out.

Table 1. Primary outcome at 24 weeks

	Change at 24 weeks		Mean difference between arms (95%)
	Liragrutide 3mg	Placebo	
% Weight loss			

11. 5.3.1 Model checking

The model for the primary outcome analysis includes an assumption that the model residuals are normally distributed. The normality of the residuals will be assessed through the construction of appropriate histograms and normal quantile-quantile plots. If such plots suggest that the normality assumption is violated, then appropriate transformations of the primary outcome variable will be considered.

The homoscedasticity of the residuals will be assessed using a scatter plot. Possible influential observations and outliers will be identified graphically or using summary statistics. Sensitivity to such influential observations and/or outliers (if present) will be considered.

12. 5.4 Secondary analyses

Secondary outcomes

Exploratory analyses will be performed for secondary outcomes. Plots of %WL at Week 2, 4, 8, 17 and 24 will be presented for a random sample of patients to explore the effect of treatment over time. Analysis of repeated measurement for %WL will be performed using random effects linear regression model including intervention group, type of surgical treatment, diabetes status, baseline values of weight, time and an interaction of intervention effect and time in the model if appropriate. The %WL outcome will be categorised into 3 categories: ≥5%, ≥10% and ≥15% and analysed using proportional odds model including intervention group, type of surgical treatment, diabetes status and baseline values of weight. If the model does not converge due to small numbers, a descriptive comparison will be carried out or a binary category will be used. Plots will also be used to show the trends of other continuous outcomes (e.g. fat, lean body mass, bone density, circulating fasted glucose, insulin, HbA1c and leptin, meal-stimulated glycaemic, gut hormone and appetite response) from baseline to the end of 24 weeks. These secondary continuous outcomes measured at

24 weeks will be analysed by fitting linear regression models, adjusting for type of surgical treatment, diabetes status and baseline values of the outcomes. Random effects models will be used to analyse repeated measures of the secondary outcomes. The normality assumption will be checked for each model. If violated, a suitable transformation/non-parametric method will be considered. All analyses will be carried out comparing the intervention and control groups as randomised using all available data (intention to treat analysis). Mean differences in each outcome will be reported with 95% confidence intervals.

Table 2. Secondary outcomes over time.

Outcome	Units / Category	Baseline	Week 4	Week 8	Week17	Week 24
%WL						
Fat						
Lean body mass						
Bone density						
Circulating fasted glucose						
Insulin						

All patients who withdraw consent will be excluded from the primary and secondary analyses from the point of withdrawal, although any data collected from such patients prior to the point of withdrawal will be included unless the reason specifies otherwise.

Non adherence and adverse events
Descriptive statistics for characteristics of patients who do not adhere to their treatment as specified in the protocol will be presented. Adverse events will be summarised using counts.

13. 5.5 Missing data/ Drop-out

Where possible, descriptive analysis of the characteristics of patients who withdraw consent will be performed and reasons for withdrawal will be described. A descriptive comparison of drop-out rates between trial arms will be undertaken using tabulations or graphs as appropriate. The characteristics of patients who drop out will be summarised where possible.

Bias due to missing data will be investigated by comparing the baseline characteristics of participants with and without missing values. Depending on the extent of missingness, the predictors of missing values will be identified using logistic regression. As part of a

supportive analysis for the primary outcome, the predictors of missingness related to the outcome will be included in the analysis model.

Imputation of missing values of the primary outcome at week 24 may be performed using the complete outcome values collected at other measurement time points if considered appropriate or using self reported weight. Predictors of missingness and the predictors included in the analysis model will be included in the imputation model. The missing values will be imputed separately for each randomised group. The handling of missing data for the analysis of the secondary outcomes will follow the same strategy if considered appropriate.

14. 5.6 Reporting

Analyses will be reported with respect to the CONSORT checklist and with any particular requirements of journals to which the results of analyses are submitted.

6. 6 Reference

[1] Pi-Sunyer, X., et al., A Randomized, Controlled Trial of 3.0 mg of Liraglutide in Weight Management. N Engl J Med, 2015. 373(1): p. 11-22.

[2] StataCorp. 2017. Stata Statistical Software: Release 15. College Station, TX: StataCorp LLC.

[3] Schulz KF, Altman DG, Moher D., CONSORT 2010 Statement: updated guidelines for reporting parallel group randomised. Trials. 2010 Mar 24;11(1):32.

7. 7 Signatures

[redacted]	[redacted]	[redacted]
Chair of DSMC (Print Name)	Chair of DSMC (Signature)	Date
[redacted]	[redacted]	[redacted]
Chair of TSC (Print Name)	Chair of TSC (Signature)	Date
