# Supplementary material for: Safety and Efficacy of Liraglutide, 3.0 mg, Once Daily vs Placebo in Patients With Poor Weight Loss Following Metabolic Surgery: The BARI-OPTIMISE Randomized Clinical Trial
Source: JAMA Surg. 2023 Jul 26;158(10):1003–11. doi: 10.1001/jamasurg.2023.2930 (PMC10372755; doi:10.1001/jamasurg.2023.2930)
Supplement: Supplement 2. — eTable 1. Analysis of primary outcome at multiple time points eTable 2. Patients that lost ≥ 5%, ≥ 10% and ≥ 15% of their body weight at 24 weeks eTable 3. Baseline values for functional capacity tests, BDI score, IWQOL-Lite score, IPAQ activity and EQ-53-3L score eTable 4. Analysis of physical function tests between baseline and week 24 eTable 5. Analysis of IPAQ questionnaire between baseline and week 24 eTable 6. Analysis of IQWOL-Lite and components between baseline and week 24 eFigure 1. Trial design eFigure 2. Analysis of change in weight at multiple time points (kg) eFigure 3. Effect of liraglutide 3.0mg once daily and placebo on categorical weight loss [file jamasurg-e232930-s002.pdf]

Supplemental Online Content

Mok J, Adeleke MO, Brown A, et al. Safety and efficacy of liraglutide, 3.0 mg, once daily vs placebo in patients with poor weight loss following metabolic surgery: the BARI-OPTIMISE randomized clinical trial. *JAMA Surg*. Published online July 26, 2023. doi:10.1001/jamasurg.2023.2930

eTable 1. Analysis of primary outcome at multiple time points

eTable 2. Patients that lost ≥ 5%, ≥ 10% and ≥ 15% of their body weight at 24 weeks

eTable 3. Baseline values for functional capacity tests, BDI score, IWQOL-Lite score, IPAQ activity and EQ-53-3L score

eTable 4. Analysis of physical function tests between baseline and week 24

eTable 5. Analysis of IPAQ questionnaire between baseline and week 24

eTable 6. Analysis of IQWOL-Lite and components between baseline and week 24

eFigure 1. Trial design

eFigure 2. Analysis of change in weight at multiple time points (kg)

eFigure 3. Effect of liraglutide 3.0mg once daily and placebo on categorical weight loss

This supplemental material has been provided by the authors to give readers additional information about their work.

© 2023 Mok J et al. *JAMA Surgery*.

Supplementary analysis

Model checking

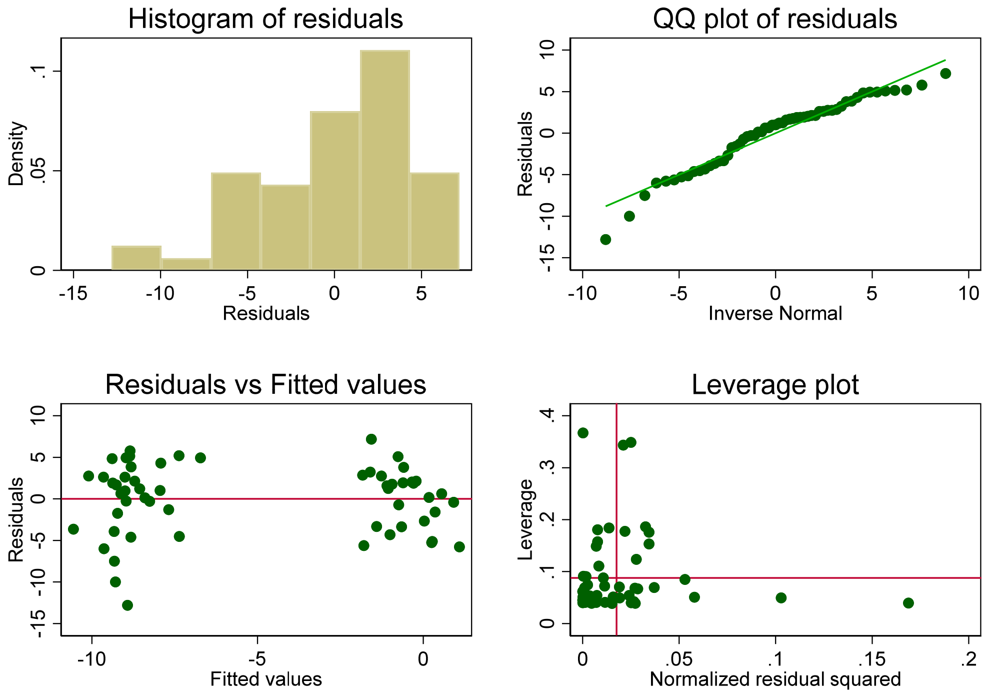

From the histogram and qq-plot, it appears that the normality assumption might not be satisfied. The residual vs fitted plot suggest that the homoscedasticity assumption might be violated because the points are more spread out around -10 than around 0. The leverage plot has heighted some influential observations. There are some observations that have high residuals, but they do not have high leverage.

Sensitivity analysis

A sensitivity analysis for influential points was carried out, observations with leverage greater than or equal to 0.1, 13 observations (5 in placebo and 8 in liraglutide arm), were removed and the primary outcome model was re-fitted, the estimate (and 95% C.I.) is -8.77 (-11.43 to -6.10).

A quantile regression model for median was fitted as a sensitivity analysis for the violation of the normality assumption of residuals in the primary analysis. The quantile regression compares the median %WL in the liraglutide and placebo arm, the estimate (and 95% C.I.) is -8.51 (-11.72 to -5.29). This implies that the adjusted difference of the median %WL in the liraglutide and placebo arms is -8.51.

A sensitivity analysis for homoscedasticity assumption is carried out by specifying a robust standard error (SE) for the primary analysis model. If the homoscedasticity assumption is not satisfied, the standard error of the estimate (and the C.I.) might be misleading, the robust SE specification could protect against this. The estimate (and 95% C.I.) after specifying robust SE is -8.03 (-10.32 to -5.73).

© 2023 Mok J et al. *JAMA Surgery*.

eTable 1: Analysis of primary outcome at multiple time points

	Placebo		Liraglutide		Adjusted mean difference* (95% CI)
	n	Mean (SD)	n	Mean (SD)	
Weight loss (%)					
Week 2	30	-0.25 (0.96)	32	-1.80 (0.96)	-1.54 (-2.83 to -0.26)
Week 4	33	-0.39 (1.31)	31	-3.44 (1.41)	-3.0 (-4.27 to -1.71)
Week 8	34	-0.44 (1.92)	32	-4.97 (2.12)	-4.45 (-5.72 to -3.19)
Week 17	30	-0.70 (2.71)	29	-7.85 (3.91)	-6.92 (-8.23 to -5.61)
Week 24	34	-0.14 (3.28)	32	-8.6 (4.96)	-8.44 (-9.71 to -7.18)

* Estimated from mixed effects model adjusting for baseline weight, type of surgery and diabetes status.

© 2023 Mok J et al. *JAMA Surgery*.

eTable 2: Patients that lost ≥ 5%, ≥ 10% and ≥ 15% of their body weight at 24 weeks

	Placebo	Liraglutide
≥ 5% weight loss	3 (8.8%)	23 (71.9%)
≥ 10% weight loss	0 (0%)	9 (28.1%)
≥ 15% weight loss	0 (0%)	4 (12.5%)

The number of patients in placebo arm that lost more than 5% of their body weight is small, so we only present the descriptive analysis. Similarly, because no patient in placebo arm lost 10% or more or 15% or more of their body weight, we cannot fit a logistic regression models for these outcomes. Regression models fitted for these outcomes will not converge.

© 2023 Mok J et al. *JAMA Surgery*.

eTable 3: Baseline values for functional capacity tests, BDI score, IWQOL-Lite score, IPAQ activity and EQ-53-3L score.

Group allocation	Placebo (n=35)	Liraglutide (n=35)
**6 Minute Walk distance (m)***	445.6 (95.7)	464.5 (89.7)
**5 Sit stand test duration (secs)****	12.2 (3.3)	13.3 (5.4)
**Hand grip test (kg)+**	35.6 (13.5)	31.6 (9.9)
**BDI Score**	13.8 (13.6)	16.3 (10)
**IWQOL-Lite total score**	55.8 (32.1)	56.3 (23.8)
**Physical function**	56.1 (25.4)	55 (25.2)
**Self esteem**	41.7 (29)	43.3 (29.4)
**Sex life** (3)	63.6 (34)	61.8 (30.9)
**Public distress**	55.1 (30.6)	63.3 (27.8)
**Work**(4)	73.2 (26.7)	71.1 (31.3)
**Number of days on vigorous activity**		
**0**	19 (54.3%)	25 (71.4%)
**1**	7 (20.0%)	2 (5.7%)
**2**	4 (11.4%)	4 (11.4%)
**3**	2 (5.7%)	4 (11.4%)
**4**	0 (0%)	0 (0%)
**5**	2 (5.7%)	0 (0%)
**6**	0 (0%)	0 (0%)
**7**	1 (2.9%)	0 (0%)
**Number of days on moderate activity**		
**0**	23 (65.7%)	19 (54.3%)
**1**	1 (2.9%)	1 (2.9%)
**2**	1 (2.9%)	5 (14.3%)
**3**	5 (14.3%)	5 (14.3%)
**4**	2 (5.7%)	1 (2.9%)
**5**	1 (2.9%)	1 (2.9%)
**6**	1 (2.9%)	0 (0%)
**7**	1 (2.9%)	3 (8.6%)
**Number of days on walking activity**		
**0**	2 (5.7%)	2 (5.7%)
**1**	3 (8.6%)	2 (5.7%)
**2**	5 (14.3%)	3 (8.6%)
**3**	1 (2.9%)	3 (8.6%)
**4**	1 (2.9%)	2 (5.7%)
**5**	8 (22.9%)	8 (22.9%)
**6**	2 (5.7%)	0 (0%)
**7**	13 (37.1%)	15 (42.9%)
**IPAQ Score (Activity level)** (5)		
**Low**	15 (42.9%)	11 (32.4%)
**Moderate**	8 (22.9%)	15 (44.1%)
**High**	12 (34.3%)	8 (23.5%)
**EQ-53-3L score**	0.6 (0.4)	0.7 (0.3)

*4 patient (3 in control arm and 1 in liraglutide arm) did not attempt the 6-minute walk test. 4 patients (1 in control arm and 3 in liraglutide arm) attempted but did not complete the 6-minute walk test. ** 3 patients (2 in control arm and 1 in liraglutide arm) did not attempt 5-sit-stand test, 4 patients in liraglutide arm did not complete 5-sit-stand test

+ the maximum weight for both hand is reported.

(3) 2 sex life scores missing (1 each arm)

(4) 1 missing work score in liraglutide arm.

(5) 1 patient in liraglutide arm removed because their measurement is an outlier according to the scoring guideline. IPAQ questionnaire was scored and categorised following the IPAQ scoring protocol [1].

© 2023 Mok J et al. *JAMA Surgery.*

eTable 4: Analysis of physical function tests between baseline and week 24

	Placebo		Liraglutide		Adjusted mean difference* (95% CI)
	n	Mean (SD)	n	Mean (SD)	
6 Minute Walk distance (m)	24	15.50 (51.28)	26	21.31 (48.57)	9.13 (-21.10 to 39.35)
5 sit stand duration (secs)	27	0.44 (3.26)	25	-1.84 (3.69)	-1.97 (-3.92 to -0.03)
Hand grip test (kg)	28	-3.71 (10.19)	30	-2.13 (4.97)	0.07 (-3.58 to 3.73)

*Estimated from linear regression model adjusting for baseline value of the secondary outcome, baseline weight, type of surgery and diabetes status.

© 2023 Mok J et al. *JAMA Surgery*.

eTable 5: Analysis of IPAQ questionnaire between baseline and week 24

	Placebo	Liraglutide	Odds ratio* (95% CI)
	n (%)	n (%)	
Activity level			1.04 (0.37 to 2.90)
Low	13 (39.4%)	8 (26.7%)	
Moderate	7 (21.2%)	11 (36.7%)	
High	13 (39.4%)	11 (36.7%)	

*Estimated from proportional odds model adjusting for baseline value of the secondary outcome, baseline weight, type of surgery and diabetes status.

© 2023 Mok J et al. *JAMA Surgery*.

eTable 6: Analysis of IQWOL-Lite and components between baseline and week 24

	Placebo		Liraglutide		Adjusted mean difference* (95% CI)
	n	Mean (SD)	n	Mean (SD)	
IWQOL-Lite total score	33	-0.93 (10.70)	32	4.98 (15.05)	7.13 (0.60 to 13.66)
Physical function	33	-0.28 (11.29)	32	5.54 (18.48)	7.54 (0.10 to 14.99)
Self-esteem	33	-3.57 (15.72)	32	4.6 (21.30)	8.77 (-0.51 to 18.06)
Sex life	32	-2.93 (20.82)	31	4.64 (28.27)	7.00 (-5.60 to 19.60)
Public distress	33	1.97 (18.45)	32	5.31 (16.80)	5.04 (-3.89 to 13.98)
Work	33	-1.52 (17.19)	31	1.21 (21.07)	3.12 (-6.43 to 12.66)

*Estimated from linear regression model adjusting for baseline value of the secondary outcome, baseline weight, type of surgery and diabetes status. Higher scores of IQWOL-Lite indicate a better quality of life.

© 2023 Mok J et al. *JAMA Surgery.*

eFigure 1: Trial design

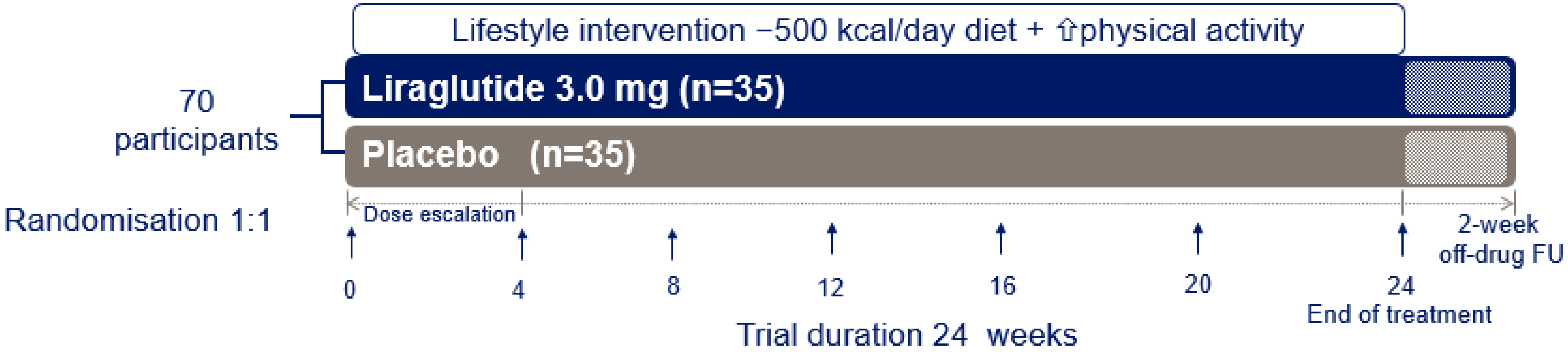

© 2023 Mok J et al. *JAMA Surgery.*

eFigure 2: Analysis of change in weight at multiple time points (kg)

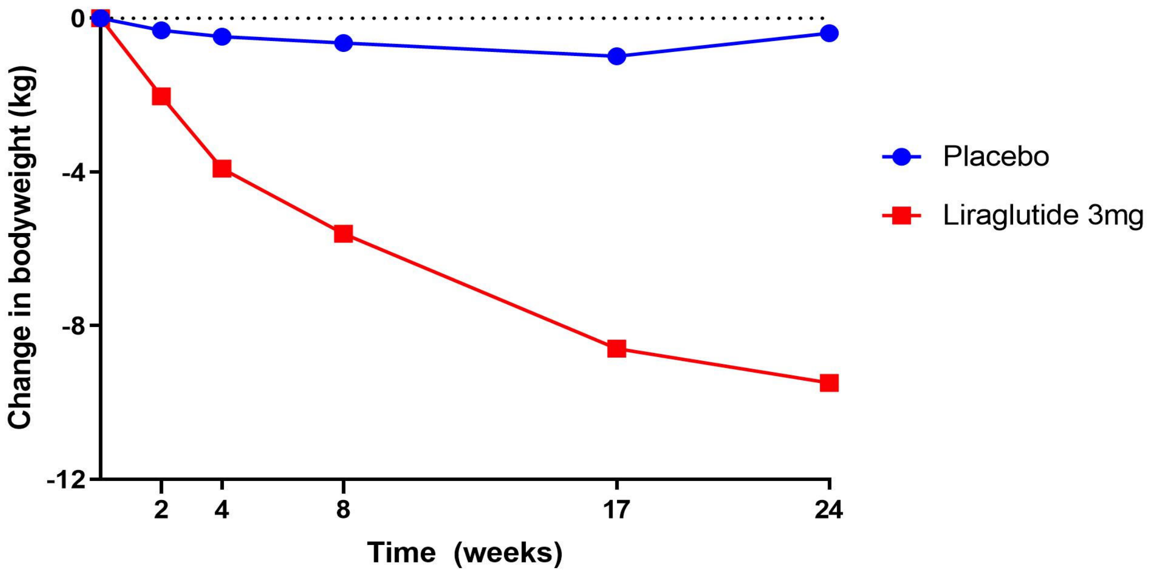

**Placebo (n)**	**35**	**30**	**33**	**34**	**30**	**34**
**Liraglutide (n)**	**35**	**32**	**31**	**32**	**29**	**32**

Observed mean body weight change in kg over time for full anlaysis set.

© 2023 Mok J et al. *JAMA Surgery.*

eFigure 3: Effect of liraglutide 3.0mg once daily and placebo on categorical weight loss

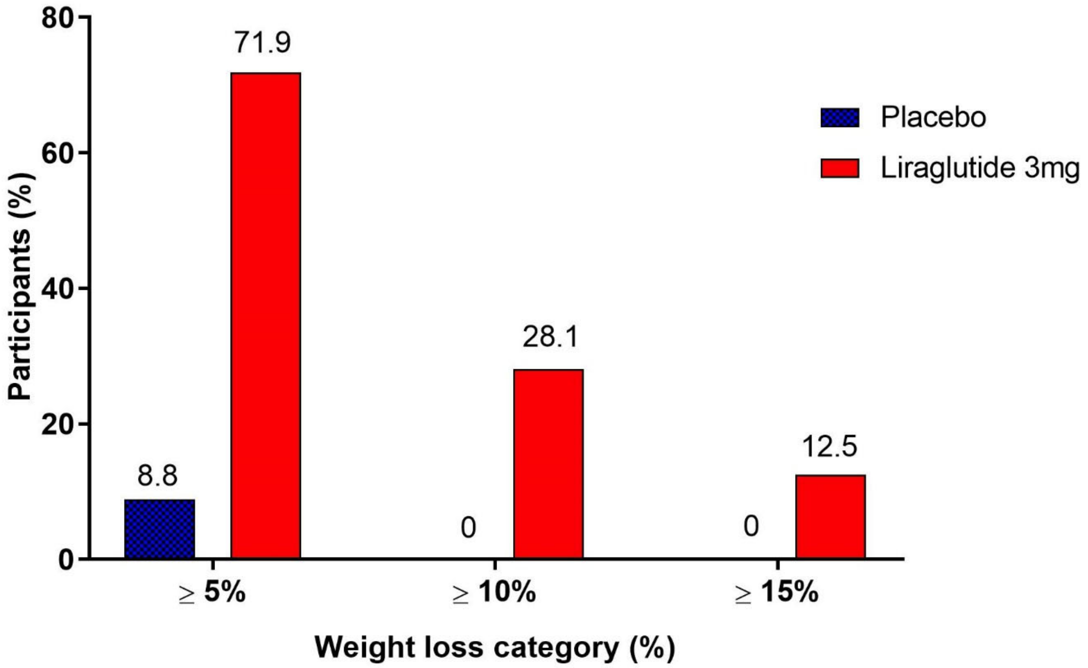

Figure illustrating categorical weight loss following treatment with liraglutide 3.0mg once daily (red bars) and placebo (blue bar), in terms of percentage of participants who achieved weight loss of ≥5%, ≥10% and ≥15% from their baseline bodyweight respectively. 8.8% of participants in the placebo group versus 71.9% in the liraglutide group achieved ≥5% weight loss. No participants from the placebo group achieved ≥10% weight loss, whereas 28.1% in the liraglutide group lost ≥10% and 12.5% ≥15% of their baseline weight.

© 2023 Mok J et al. *JAMA Surgery*.
