# Supplementary material for: Safety and Efficacy of Liraglutide, 3.0 mg, Once Daily vs Placebo in Patients With Poor Weight Loss Following Metabolic Surgery: The BARI-OPTIMISE Randomized Clinical Trial
Source: JAMA Surg. 2023 Jul 26;158(10):1003–11. doi: 10.1001/jamasurg.2023.2930 (PMC10372755; doi:10.1001/jamasurg.2023.2930)
Supplement: Supplement 3. — Data sharing statement [file jamasurg-e232930-s003.pdf]

Data Sharing Statement

Mok. Safety and Efficacy of Liraglutide, 3.0 mg, Once Daily vs Placebo in Patients With Poor Weight Loss Following Metabolic Surgery. *JAMA Surg*. Published July 26, 2023. doi:10.1001/jamasurg.2023.2930

Data
Data available: Yes
Data types: Deidentified participant data
How to access data: Post-publication
When available: With publication

Supporting Documents
Document types: Other (please specify)
Additional Information: Supplementary appendix with trial protocol, statistical analysis plan and secondary analyses
How to access documents: With submission as appendix
When available: With publication

Additional Information
Who can access the data: Study data though Pi with data sharing agreement. A supplementary appendix with the trial protocol, statistical analysis plan and secondary analyses has been submitted.
Types of analyses: As per data sharing agreement.
Mechanisms of data availability: With investigator approval.
